# Evolutionary genomics reveals variation in structure and genetic content implicated in virulence and lifestyle in the genus *Gaeumannomyces*

**DOI:** 10.1186/s12864-025-11432-0

**Published:** 2025-03-12

**Authors:** Rowena Hill, Michelle Grey, Mariano Olivera Fedi, Daniel Smith, Gail Canning, Sabrina J. Ward, Naomi Irish, Jade Smith, Vanessa E. McMillan, Jess Hammond, Sarah-Jane Osborne, Gillian Reynolds, Ellie Smith, Tania Chancellor, David Swarbreck, Neil Hall, Javier Palma-Guerrero, Kim E. Hammond-Kosack, Mark McMullan

**Affiliations:** 1 Earlham Institute, Norwich Research Park, Norwich, NR4 7UZ UK; 2 Rothamsted Research, Harpenden, AL5 2JQ UK; 3 John Innes Centre, Norwich, Norfolk NR4 7UH UK; 4 NIAB, 93 Lawrence Weaver Road, Cambridge, CB3 0LE UK; 5 AHDB, Siskin Parkway East, Middlemarch Business Park, Coventry, CV3 4PE UK; 6 Crop Science Centre, Department of Plant Sciences, University of Cambridge, 93 Lawrence Weaver Road, Cambridge, CB3 0LE UK; 7 School of Biological Sciences, University of East Anglia, Norwich, NR4 7TJ UK; 8 Research Institute of Organic Agriculture Fibl, Frick, 5070 Switzerland

**Keywords:** Take-all, Magnaporthales, Plant pathogen, Endophyte, Starships

## Abstract

*Gaeumannomyces tritici* is responsible for take-all disease, one of the most important wheat root threats worldwide. High-quality annotated genome resources are sorely lacking for this pathogen, as well as for the closely related antagonist and potential wheat take-all biocontrol agent, *G. hyphopodioides*. As such, we know very little about the genetic basis of the interactions in this host–pathogen–antagonist system. Using PacBio HiFi sequencing technology we have generated nine near-complete assemblies, including two different virulence lineages for *G. tritici* and the first assemblies for *G. hyphopodioides* and *G. avenae* (oat take-all). Genomic signatures support the presence of two distinct virulence lineages in *G. tritici* (types A and B), with A strains potentially employing a mechanism to prevent gene copy-number expansions. The CAZyme repertoire was highly conserved across *Gaeumannomyces*, while candidate secreted effector proteins and biosynthetic gene clusters showed more variability and may distinguish pathogenic and non-pathogenic lineages. A transition from self-sterility (heterothallism) to self-fertility (homothallism) may also be a key innovation implicated in lifestyle. We did not find evidence for transposable element and effector gene compartmentalisation in the genus, however the presence of *Starship* giant transposable elements may contribute to genomic plasticity in the genus. Our results depict *Gaeumannomyces* as an ideal system to explore interactions within the rhizosphere, the nuances of intraspecific virulence, interspecific antagonism, and fungal lifestyle evolution. The foundational genomic resources provided here will enable the development of diagnostics and surveillance of understudied but agriculturally important fungal pathogens.

**Supplementary Information:**

The online version contains supplementary material available at 10.1186/s12864-025-11432-0.

## Introduction

*Gaeumannomyces* is a broadly distributed genus of *Poaceae* grass-associated root-fungi [1], best known for the species *Gaeumannomyces tritici* (*Gt*) which causes take-all disease, the most serious root disease of wheat [2]. *Gaeumannomyces* is a comparatively understudied genus despite belonging to the *Magnaporthales*, an economically important order of pathogens including the rice and wheat blast fungus *Pyricularia oryzae* (syn. *Magnaporthe oryzae* [3]). This is perhaps due to a historical research bias towards above-ground pathogens, in part simply due to the fact that characteristic symptoms of root pathogen diseases are hidden from view [4, 5]. Recently the rhizosphere has received more research attention as its key role in plant health and productivity has become apparent [6]. There have also been considerable difficulties in producing a reliable transformation system for *Gt*, preventing gene disruption experiments to elucidate function [7].

Although genetic studies of *Gt* have been limited, single-locus phylogenetic analyses of *Gt* have consistently recovered two distinct lineages within the species [8], which we will refer to using the ‘A/B’ characterisation established by Freeman et al. [9] based on *ITS2* polymorphism. Although very little is known about the dynamics of these two lineages, each is found across the world and both lineages persistently co-occur in the same field, prompting the suggestion that the two lineages may actually be cryptic species [2, 8]. Although variation within lineages is high, there is also some evidence that type A strains are more virulent [10–12], which is a major impetus for improving our understanding of these two lineages. The sister species to *Gt*, *G. avenae* (*Ga*), has infrequently been reported to infect wheat, but is not the predominant agent of wheat take-all, and is distinguished by the fact that production of avenacinase enables *Ga* to infect oat roots [13, 14].

 The order* Magnaporthales* is also home to several commensal and/or mutualistic fungi [15], including those with the potential to inhibit take-all [16]. For instance, *G. hyphopodioides* (*Gh*) — a species closely related to *Gt* that also grows on wheat roots— is not only non-pathogenic, but actually capable of suppressing take-all to varying degrees [17]. It is now apparent that prior *Gh* colonisation primes the host plant’s immune response [18], a mechanism that has been reported in various other plant–microbe interactions associated with disease prevention [19, 20]. This has prompted interest in *Gh* as a potential biocontrol agent, for instance by adding *Gh* inoculant to wheat seedstock via seed coating [21] and/or selecting for wheat cultivars that support enhanced levels of *Gh* root system colonisation [17]. Novel disease prevention approaches for take-all are especially desirable as up to 30% of *Gt* strains are found to be naturally resistant to the seed-dressing fungicide routinely used to treat take-all, silthiofam [9].

Understanding the genetic machinery underpinning virulence and lifestyle in *Gaeumannomyces* has previously been hampered by a lack of genomic data. Prior to the present study, a single annotated *Gt* assembly (strain R3-111a-1), sequenced using the 454 platform, was available on NCBI (accession GCF_000145635.1) [22] – one other more recent PacBio assembly has been released for the same strain, but remains unannotated (GCA_016080095.1). This scarcity of genomic resources has not only limited our understanding of the genetics of the system, but also accounts for a lack of molecular diagnostics for take-all. Given the increase in research activities since 2005 following the production of genomic resources for *P. oryzae* [23, 24], we are optimistic that providing similar high-quality assemblies for *Gaeumannomyces* species will bolster research efforts in the global take-all community.

Here, we have addressed the gap in genomic resources for *Gaeumannomyces* by generating near-complete assemblies for nine strains, including both type A and B *Gt* lineages and the first assemblies for *Gh* and *Ga*. Using an evolutionary genomics approach, we identified variation in structure as well as gene features known to be involved in plant-fungal interactions — candidate secreted effector proteins (CSEPs), carbohydrate-active enzymes (CAZymes) and biosynthetic gene clusters (BGCs) — to address the questions: (1) Are there genomic signatures distinguishing *Gt* A/B virulence lineages? (2) How do gene repertoires differ between pathogenic *Gt* and non-pathogenic *Gh*? and (3) Is there evidence of genome compartmentalisation in *Gaeumannomyces*? In the process of doing so, we also identified giant cargo-carrying transposable elements belonging to the recently established *Starship* superfamily [25].

## Results

### Evidence of greater take-all severity caused by *G. tritici* type A strains

**Fig. 1:**
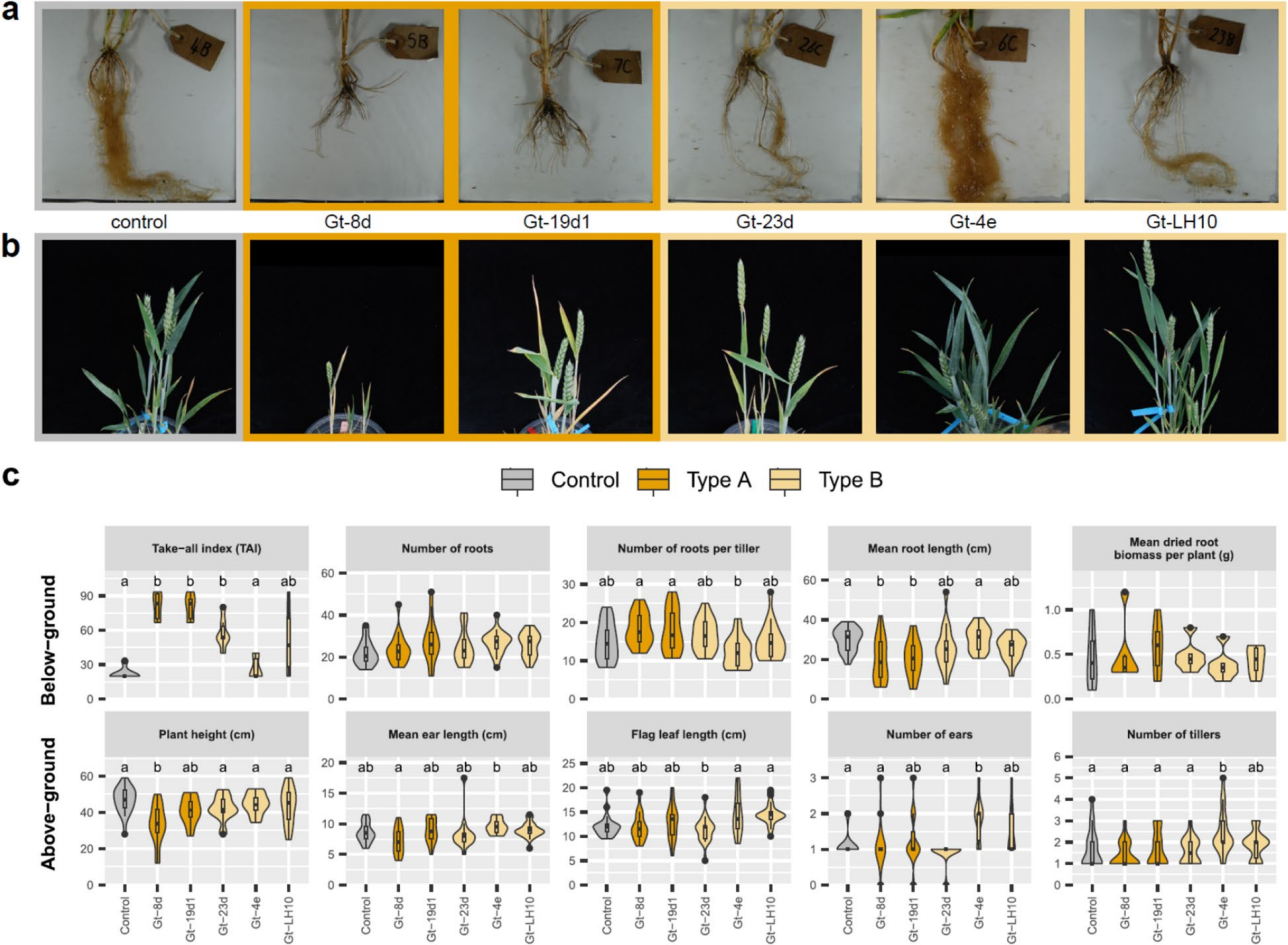
Intraspecific variation in *Gaeumannomyces tritici* (*Gt)* virulence assessed from inoculation of wheat plants. Representative photos of wheat roots (**a**) and above-ground features (**b**) following inoculation treatment. Inoculated strains from left to right: no *Gt* (control), Gt-8d, Gt-19d1, Gt-23d, Gt-4e and Gt-LH10. **c** Box and violin plots showing the impact of the five *Gt* strains sequenced in this study on above- and below-ground characteristics in winter wheat. Control, *Gt* type A and type B groups are indicated by different colours. Strains with a significant mean difference for the characteristic as calculated by either the Tukey HSD or Games-Howell test are shown by letter groups above the box and violin plots

As the five *Gt* strains sequenced in this study included representatives of both the type A and B lineages, we performed a season long inoculation experiment to determine the relative capacity for each strain to cause take-all disease symptoms. From general visual inspection, inoculation of *Gt*A strains into the highly susceptible winter wheat cultivar Hereward resulted in notably depleted roots compared to a control and, to a lesser extent, *Gt*B strains (Fig. 1a). Inoculation with *Gt*A strains also resulted in a visible reduction of overall plant size compared to the control, while *Gt*B-inoculated plants were less easily distinguished from the control (Fig. 1b). Although above- and below-ground characteristics of wheat varied depending on *Gt* strain, our statistical analysis showed that the *Gt*A strains had a greater capacity to reduce plant height and reduce root length, and both *Gt*A strains consistently produced the greatest root disease symptoms, i.e. highest Take-all Index (TAI) scores [26] (Fig. 1c). Furthermore, five out of six wheat plants that died during the experiment were inoculated with *Gt*A strains. Several characteristics were inconsistently affected by *Gt* inoculation, including mean floral spike (ear) length; dried root biomass; number of roots; and number of roots per tiller.

### Nine near-complete *Gaeumannomyces* assemblies, including first genome assemblies for *G. avenae* and *G. hyphopodioides*

We used PacBio HiFi sequencing technology to produce highly contiguous genome assemblies for five *Gt*, two *Gh* and two *Ga* strains (see Supplemental Fig. S1 for a schematic summarising the bioinformatics workflow). All nine assembled genomes had N50 values of more than 4 Mb (Supplemental Table S1), a 100-fold increase on the N50 of the existing annotated *Gt* RefSeq assembly (NCBI accession GCF_000145635.1). In addition, transcriptomes were sequenced for all nine strains to inform gene prediction, and between 22–29% of annotated gene models had two or more isoforms across all strains (Supplemental Fig. S2). Contigs corresponding to mitochondrial genomes were identified from all assemblies (Supplemental Table S1), however circularisation was only successfully detected for two strains (Gt-23d and Ga-CB1). For most strains the overall mitogenome size, GC content and number of genes fell within the expected range for ascomycetes [27], however the mitogenome assembly for Gt-LH10 is likely incomplete, as it was a third of the size of the other *Gt*B strains, and only had 23 genes annotated compared to the 38–40 genes found for all other strains (Table S1).

**Fig. 2:**
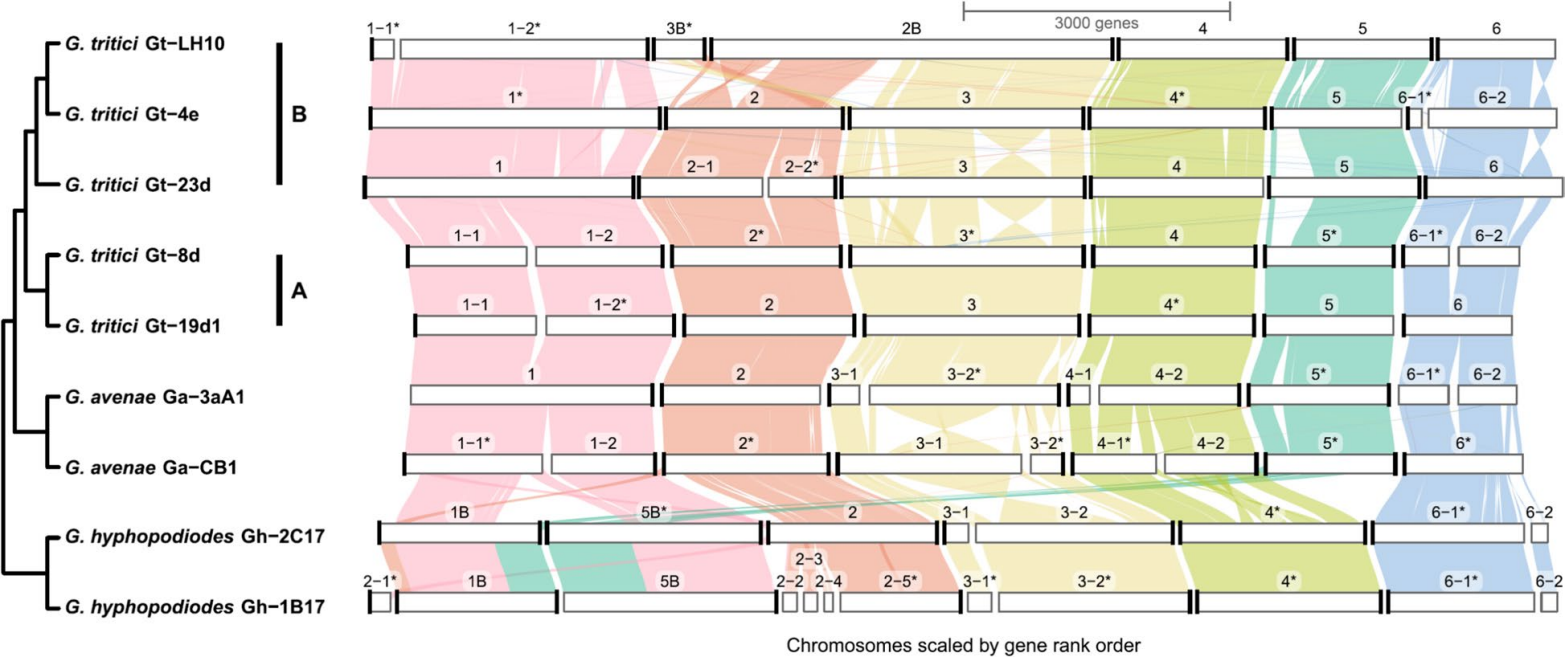
GENESPACE plot showing synteny across the nine *Gaeumannomyces* strains. A/B lineages are indicated for *G. tritici* strains. Only contigs with annotated gene models are considered by GENESPACE. Fragments are labelled with numbers corresponding to pseudochromosomes, and an asterisk indicates that a fragment was inverted in the visualisation. Black bars on the ends of fragments indicate telomeres predicted using Tapestry

Combined GENESPACE [28] and telomere prediction results suggested six chromosomes for *Gaeumannomyces* (Fig. 2), one less than *P. oryzae* [24]. Telomere-to-telomere sequences were assembled for at least five out of six pseudochromosomes for most strains. By plotting GC content alongside transposable element (TE) and gene density, we also identified AT- and TE-rich but gene-poor regions, which are putative candidates for centromeres (Supplemental Fig. S3). Some of these regions additionally correspond well with points of fragmentation in other strains, presumably due to the difficulties associated with assembly of such highly repetitive regions. Other than these occasional splits into two fragments, in most cases pseudochromosomes were entire, the exception being Gh-1B17 pseudochromosome 2 which was fragmented across five contigs.

Both *Gt*A and, to a slightly lesser degree, *Gt*B were broadly syntenic across whole pseudochromosomes, with the exception of a major chromosomal translocation between pseudochromosomes 2 and 3 in Gt-LH10 (Fig. 2). Visualisation of the spanning reads and coverage across the regions of the apparent translocation suggests the depicted arrangement is correct and not an artefact due to misassembly (Supplemental Fig. S4a), moreover there was no evidence of a block of repeats consistent with a telomere anywhere but at the ends of the pseudochromosomes (Supplemental Fig. S4c). *Ga* was also largely syntenic with *Gt*, although there were a number of inversions in Ga-CB1 pseudochromosome 3 (Fig. 2). The more distantly related *Gh* showed chromosomal translocations involving pseudochromosomes 1, 2 and 5, which were again supported by spanning reads and the absence of intrachromosomal telomeric repeats (Supplemental Fig. S4b, c).

### No evidence for significant colocalisation of transposable elements and effectors

**Fig. 3:**
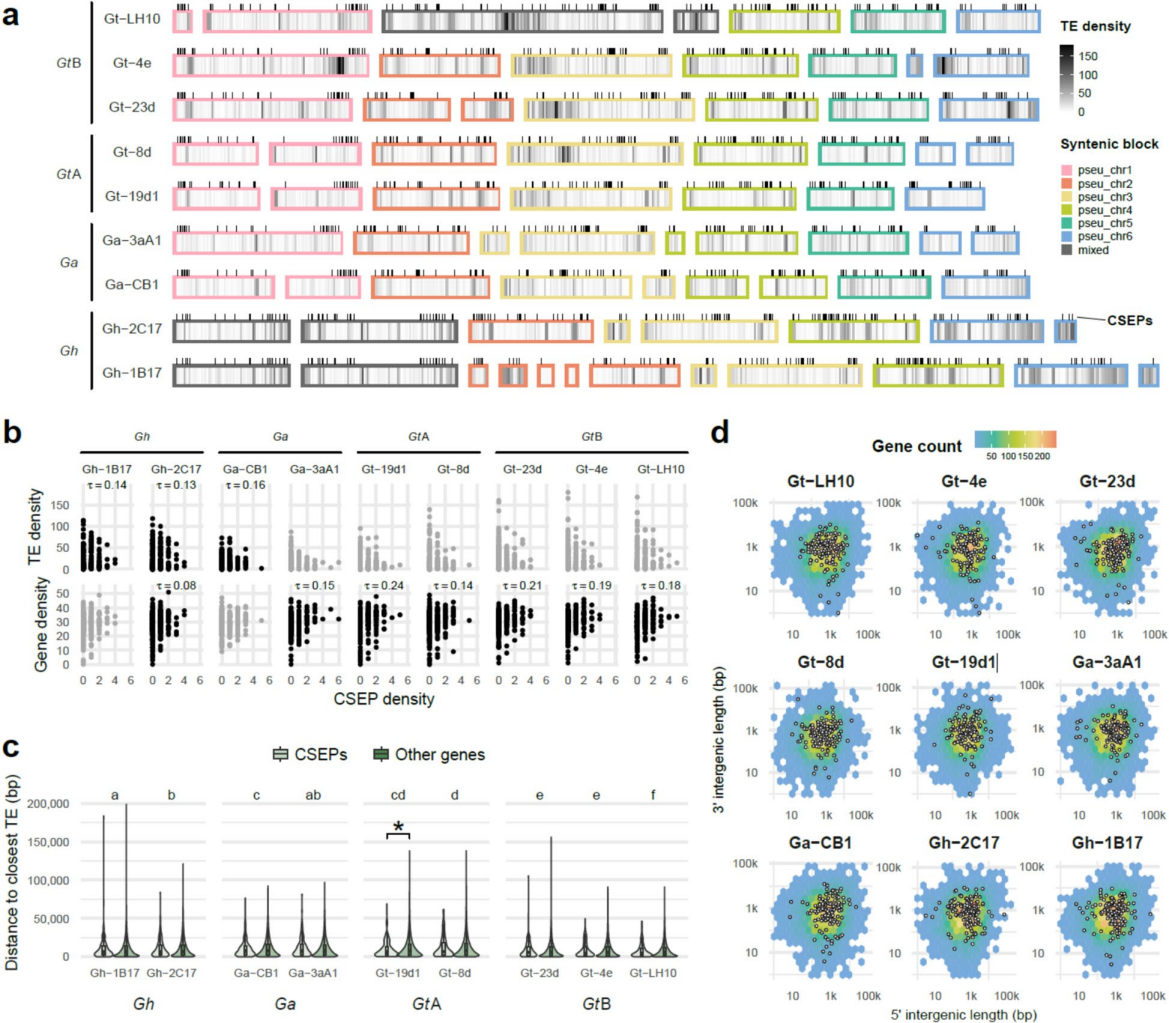
The relationship between candidate secreted effector proteins (CSEPs) and transposable elements (TEs) in *Gaeumannomyces*. **a** TE density (per 100,000 bp) and the location of CSEPs (black ticks) across fragments. Fragments are ordered syntenically according to GENESPACE (Fig. 2). **b** Scatterplot showing the relationship between CSEP density versus TE and gene density (per 100,000 bp). Significant correlation is indicated with Kendall’s tau (τ) and black points, while strains with no significant correlation are in grey. **c** Box and violin plots showing the distance of genes to the closest TE, with CSEPs and other genes distinguished by colour. An asterisk indicates where a Wilcoxon rank sum test found the mean TE distance to be significantly different for CSEPs versus other genes within an individual strain. Strains with a significant difference in mean gene-TE distance (regardless of CSEP status) as calculated by the Games-Howell test are shown by different letter groups above the plots. **d** Intergenic distances of all genes for each strain, coloured by gene density. The black outlined white points indicate CSEP genes

Compartmentalisation of effectors within genomic regions enriched in transposable elements (TEs) has previously been reported for various fungal phytopathogens [29]. In all the *Gaeumannomyces* strains sequenced here, however, we did not observe that predicted CSEPs were more likely to occur in regions of high TE density (Fig. 3a). We found a weak significant positive correlation between CSEP density and TE density for a minority of strains, however the scatterplots were unconvincing (Fig. 3b). CSEP density was more frequently found to significantly correlate with gene density, although this was still only a weak association (Fig. 3b), but the association of CSEPs with gene density was also supported by the fact that CSEPs were localised near the centre of a single hot spot of intergenic distances (Fig. 3d). For all but one strain, there was no significant difference in mean distance to closest TE for CSEPs versus other genes (Fig. 3c). For strain Gt-19d1, the mean distance from a CSEP to the closest TE was marginally lower (10,036 bp) than for other genes (12,565 bp), which permutation analysis confirmed was closer than expected based on the overall gene universe (p = 0.03), although this only remained significant for pseudochromosomes 2 and 6 when testing pseudochromosomes separately (Supplemental Fig. S5a). Individual pseudochromosomes for other strains also had lower than expected CSEP–TE distances, but with low z-scores (a proxy for strength) across the board. Comparing across strains, mean gene–TE distance was significantly different both within and between lineages, and lowest in *Gt*B (Fig. 3c). Within *Gt*B, Gt-LH10 had significantly lower mean gene–TE distance, and the same strain has also undergone an apparent expansion in total number of TEs compared to all other strains (Supplemental Fig. S6).

Although CSEPs were not broadly colocalised with TEs, we did observe that they appeared to be non-randomly distributed in some pseudochromosomes (Fig. 3a). Permutation analyses confirmed that overall CSEPs were significantly closer to telomeric regions in all strains (p = < 0.008), although by testing pseudochromosomes separately we found that this pattern varied across the genome (Supplemental Fig. S5b). CSEPs on pseudochromosomes 1, 2 and 5 were consistently closer to telomeric regions, whereas for pseudochromosomes 3 and 4 CSEPs were no closer than expected based on the gene universe. CSEPs were also closer to telomeres in pseudochromosome 6, but only in *Gt* strains.

Using a phylogenetically-informed permutational multivariate analysis of variance (PERMANOVA) method [30] to identify associations between repeat family variance and lifestyle, we found that there was a relatively high level of variance described by lifestyle (23%) (Supplemental Fig. S6).

### Core gene content in *Gaeumannomyces*

**Fig. 4:**
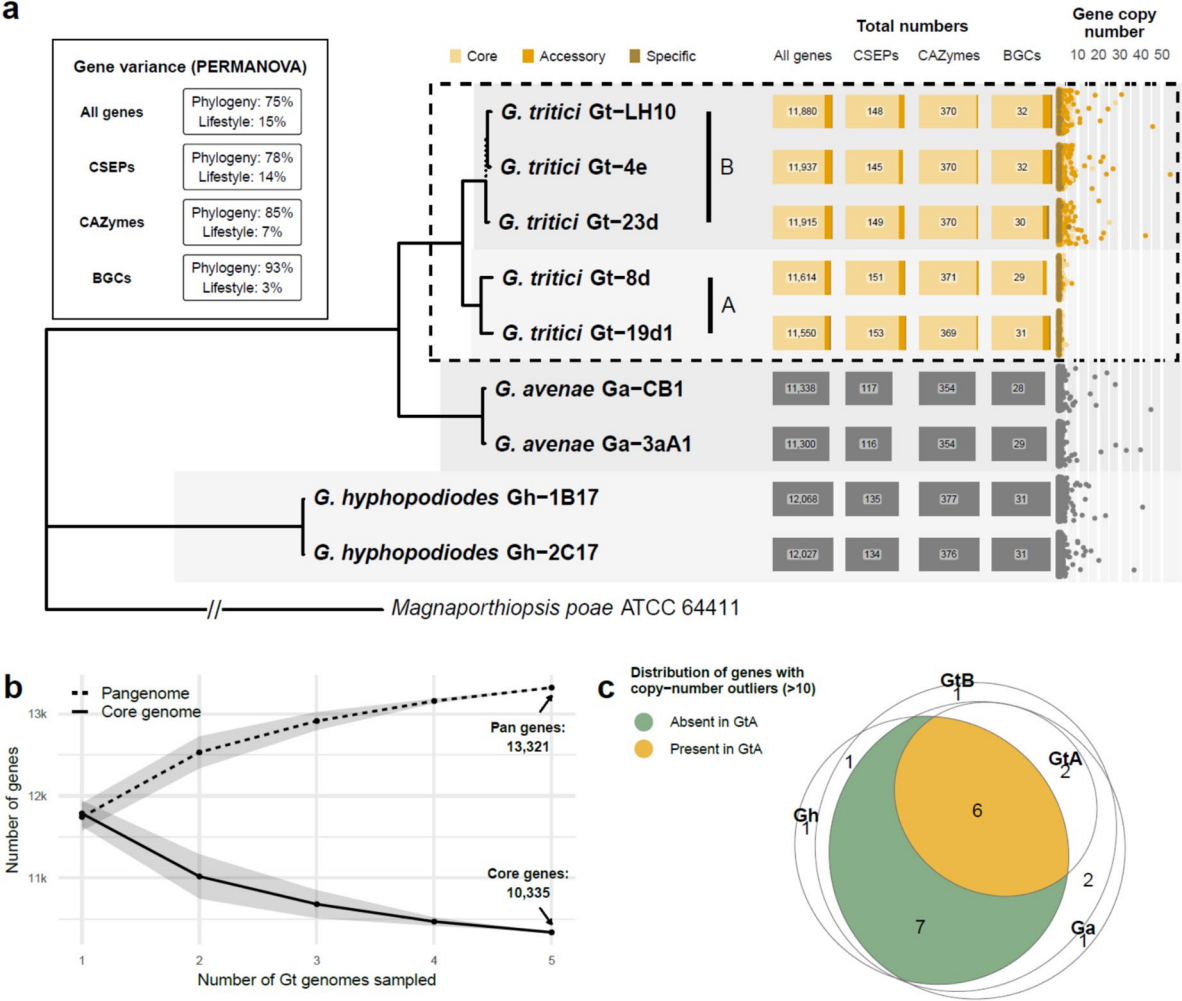
Summary of predicted gene content for the *Gaeumannomyces* strains reported in this study. **a** Number of total genes, candidate secreted effector proteins (CSEPs), carbohydrate-active enzymes (CAZymes) and biosynthetic gene clusters (BGCs) for each *Gaeumannomyces* strain. The A/B lineages are indicated for *Gaeumannomyces tritici* (*Gt*) strains. The dashed line in the phylogeny indicates bootstrap support < 70 found within the *Gt*B lineage (see Supplemental Fig. S14b for the full genome-scale *Gaeumannomyces* species tree). *Gt* gene content (within dashed box) is categorised as core (present in all strains), accessory (present in at least two strains) and specific (present in one strain). The lefthand inset box shows the results of PERMANOVA statistical tests which calculate the descriptive power of relatedness (phylogeny) versus lifestyle categorisation (*Gt* and *G. avenae* as pathogenic in wheat, *G. hyphopodioides* as non-pathogenic) on gene variance. Gene copy-number is shown on a scatterplot to the right, with points jittered vertically to improve visualisation. **b** Accumulation curves of pan and core genes for the *Gt* genomes [34]. **c** Euler diagram summarising whether high copy-number genes in each lineage are present but in low copy-number in *Gt*A, or completely absent

The total number of genes was relatively similar for all strains, although, as indicated in Fig. 2, *Gt*B and *Gh* strains had 3–6% more genes than *Gt*A or *Ga* (Fig. 4a). *Gt*A and *Gt*B had a very similar number of CSEPs, CAZymes and BGCs, however, and more CSEPs than either *Ga* or *Gh*. Almost all total genes, CSEPs and CAZymes were core in *Gt*, while there was a greater proportion of BGCs that were accessory due to lineage specific differences between the type A and B strains. From a pangenome perspective, the core gene content for *Gt* from sampling these five strains amounted to ~ 10,000 genes (Fig. 4b), which equates to ~ 88% of genes per strain being core, consistent with reports in other fungi [31]. The majority of BUSCO genes found to be missing in the assemblies were missing from all strains (Supplemental Fig. S7), suggesting that they are not present in the genus, rather than being missed as a result of sequencing or assembly errors. Three of these 18 missing core genes belonged to the *Snf7* family, which is involved in unconventional secretion of virulence factors in fungi [32], and is essential for pathogenicity in *P. oryzae* [33]. The next greatest set of missing BUSCOs (8) also seemed to be lineage specific – i.e. missing in *Gh* but present in *Gt*/*Ga* (Supplemental Fig. S7).

The avenacinase gene required for virulence on oat roots [13, 14] was identified in all strains in a conserved position on pseudochromosome 4 (Supplemental Fig. S8a). Two mating-type (MAT) loci were identified in *Gt* and *Ga*, with homologues of *Pyricularia grisea MAT1-1* and *MAT1-2* idiomorphs located in conserved but unlinked positions on pseudochromosomes 2 and 3, while only one MAT locus and idiomorph, *MAT1-1*, was identified in *Gh* on pseudochromosome 3 (Supplemental Fig. S9).

### Similarities and differences in effectors and secondary metabolite production potential between pathogenic and non-pathogenic *Gaeumannomyces* species

The number of predicted BGCs ranged from 33 to 38 per strain, which is consistent with many other ascomycete fungi [35–37]. Using the aforementioned phylogenetically-informed PERMANOVA method [30] to identify associations between gene variance and lifestyle, we found BGCs to be at the higher end of variance described purely by ancestry, 86% compared to 75%–85% for all genes, CSEPs and CAZymes (Fig. 4a). BGC variance described by lifestyle (10%) was slightly higher than for CAZymes (7%), but lower than for all genes (17%) and CSEPs (14%). CAZymes that are known to act on plant cell wall substrates were highly conserved across the genus, and there were highly similar numbers of each CAZyme family across all strains (Supplemental Fig. S10a). The only discernible pattern was marginally more copies of GH55 and GH2 (hemicellulose and pectin) in *Gh* versus the other lineages.

In total, 9% of CSEP genes could be attributed to a known gene in the Pathogen-Host Interactions database (PHI-base) [38], most of which only had one copy in all strains (Supplemental Fig. S10b). Sixteen of the 19 ‘named’ CSEPs have been associated with virulence via reverse genetics experiments, including five from *P. oryzae* infecting *Oryza sativa* (rice) — *MHP1* (ID PHI:458); *MoAAT* (PHI:2144); *MoCDIP4* (PHI:3216); *MoHPX1* (PHI:5188); and *MoMAS3* (PHI:123,065). The latter two were assigned to genes that were only present in *Gh*, although a separate gene present in *Gt*B was also characterised as *MoHPX1*. Six CSEPs in total were present in all lineages except *Gh* or vice versa. *PBC1*, also a CAZyme, the disruption of which causes complete loss of pathogenicity of *Pyrenopeziza brassicae* in *Brassica napus*, was present in *Gt* and *Ga* but not *Gh*. While *PBC1* was absent in *Gh*, all *Gaeumannomyces* strains did have some genes belonging to the same CAZyme family (CE5; Supplemental Fig. S10a). We opted to use a conservative CSEP prediction approach (Supplemental Fig. S1b) including a final step which required a consensus that genes are ‘effector-like’ according to multiple EffectorP versions [39–41]. While a stringent approach like this does risk discarding real CSEPs, we found that removal of this last step in the workflow decreased the proportion of CSEPs found to be strain-specific or accessory in *Gt* (on average 8% versus 11% for conservative set) and did not change the statistical significance of the TE-CSEP association analyses performed with the conservative set. In the PERMANOVA, using a more liberal CSEP set also decreased the signal of lifestyle (11% versus 14% for conservative set).

The BGC families were predominantly classified as type 1 polyketide synthases (PKSI), nonribosomal peptide synthetases (NRPS) and fungal ribosomally synthesised and post-translationally modified peptides (RiPPs) (Supplemental Fig. S10c). As suggested by the PERMANOVA results, presence-absence of each BGC corresponded strongly with species/lineage, with sixteen BGCs that were present or absent in *Gh* versus other lineages, including two indole BGCs only found in *Gh* (Supplemental Fig. S10c). Five other BGCs had similarity to known clusters in the MIBiG repository [42]: the 1,8‐dihydroxynaphthalene (DHN) melanin BGC from *Pestalotiopsis fici* (MIBiG ID BGC0002161) which was present in all taxa (Supplemental Fig. S11a); the nectriapyrone BGC from *Pyricularia oryzae* (BGC0002155) which was present in all taxa (Supplemental Fig. S11b); the clavaric acid BGC from *Hypholoma sublateritium* (BGC0001248) which was present in all lineages (Supplemental Fig. S11c); the dichlorodiaporthin BGC from *Aspergillus oryzae* (BGC0002237) which was absent in *Gh* (Supplemental Fig. S11d); and the equisetin BGC from *Fusarium heterosporum* (BGC0001255) which was also absent in *Gh* (Supplemental Fig. S11e).

### Gene copy-number reduction in *G. tritici* type A

*Gt*B, *Ga* and *Gh* all had high copy-number (HCN) gene outliers (> 10 copies) that were absent in *Gt*A (Fig. 4a). These 22 HCN genes were duplicated both within and across pseudochromosomes (Supplemental Fig. S12a). GO term enrichment analyses found various terms to be significantly overrepresented amongst the HCN genes, namely: vacuolar proton-transporting V-type ATPase complex assembly (Gh-1B17, Fisher’s exact test, p = 0.01); ubiquinone biosynthetic process (Gh-2C17, p = 0.01); golgi organisation (Ga-CB1, p = 0.03); mRNA cis splicing, via spliceosome (Gt-4e, p = 0.03); mitochondrial respiratory chain complex I assembly (Gt-4e, p = 0.05); proton-transporting ATP synthase complex assembly (Gt-LH10, p = 0.03); and protein localisation to plasma membrane (Gt-LH10, p = 0.03). Visualising the location of the HCN genes across the genomes (Supplemental Fig. S13) showed them to vary in terms of distribution — from relatively localised to broadly expanded — and in terms of multi-lineage versus lineage specific expansions. HCN genes were also significantly closer to TEs compared to other genes (Supplemental Fig. S12b).

Interestingly, of the 22 HCN genes, six that were shared among all species were also present in at least one *Gt*A strain but at low copy-number, while seven genes were completely absent in *Gt*A (Fig. 4c). In total, nine genes that were HCN in at least one other lineage had low-copy orthologues in *Gt*A. Moreover, these were mostly present in just one strain within the type A lineage (Gt-8d), clustered in a ~ 1 Mbp region on pseudochromosome 3 (Supplemental Fig. S12c). This region was flanked by repetitive regions that have been subjected to repeat induced point mutation (RIP), as measured by the composite RIP index (CRI) [43], although the region had average CRI of −0.3 compared to an average CRI of −0.5 for the whole pseudochromosome. Average genome-wide RIP levels were highest in *Gt*A and *Gh* (13.8% and 13.6% of the genome RIP’d, respectively), compared to *Gt*B (10.8%) and *Ga* (12.4%) (Supplementary Table S1).

### *Gaeumannomyces* genomes contain *Starship* giant transposable elements

**Fig. 5:**
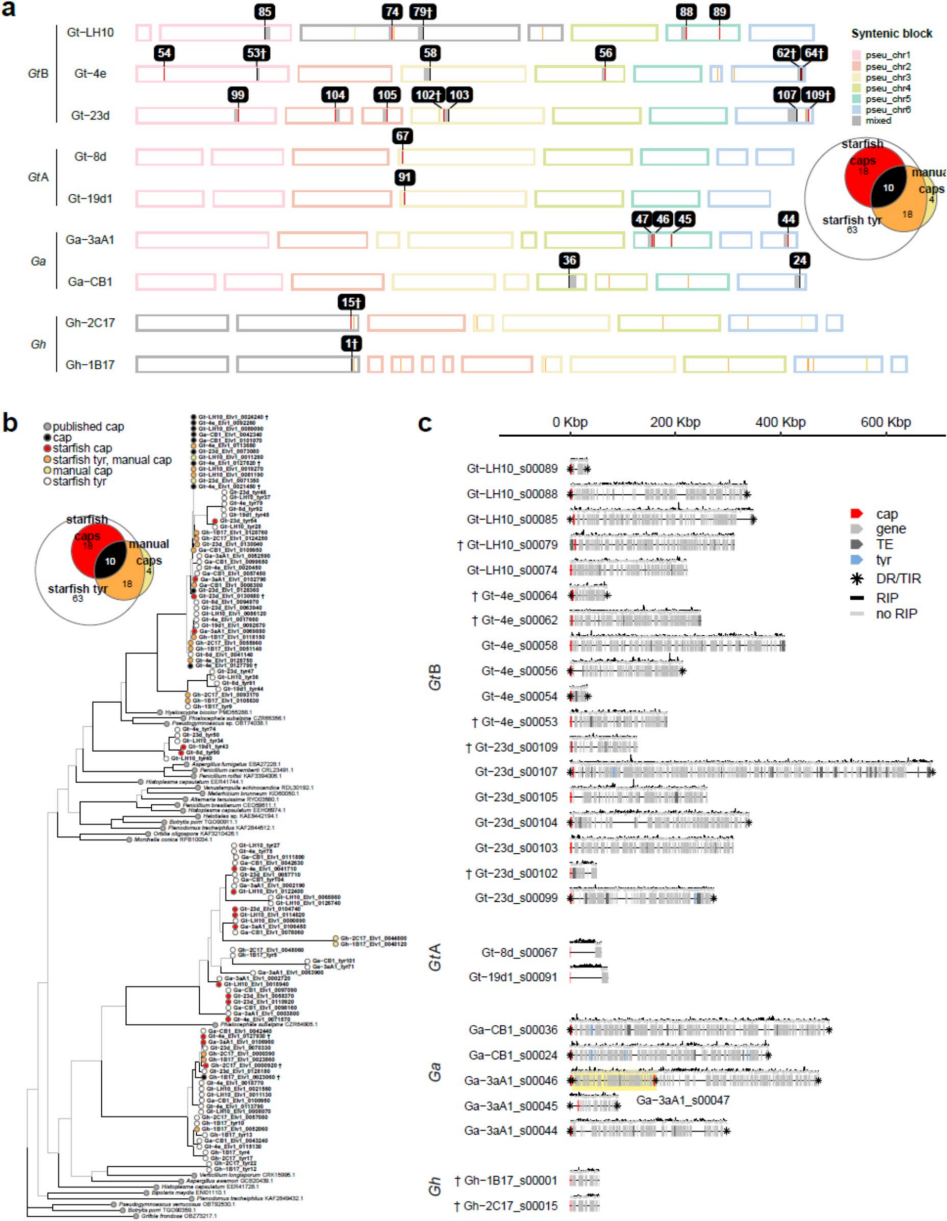
*Gaeumannomyces* genomes contain *Starship* giant transposable elements. Daggers (†) flag elements which may be false positives based on manual inspection. **a** Location of *Starship* mobile element captain genes, with colour distinguishing whether genes were identified manually or using starfish (see inset euler plot). Grey blocks indicate associated cargo genes identified by starfish. Numbering corresponds to element IDs shown in Fig. 5c. **b** Gene tree of *Starship* ‘captain’ genes, including captains and other tyrosine recombinases identified from our assemblies via starfish, captain homologues identified via blastp, and previously published captain genes. **c** A summary of the *Starship* elements identified by starfish with the composite RIP index (CRI) shown above each element. The yellow highlight distinguishes a nested element. cap = captain gene, DR = direct repeat, RIP = repeat-induced point mutation, TE = transposable element gene, TIR = terminal inverted repeat, tyr = tyrosine recombinase gene

All nine *Gaeumannomyces* strains were found to contain at least one giant TE belonging to the *Starship* superfamily of giant cargo-carrying TEs [25], identified using the tool starfish [44]. Currently the most reliable identifying feature of *Starships* is a single ‘captain’ gene – a tyrosine recombinase gene containing a DUF3435 domain which is found in the first position of each *Starship* and directs the mobilisation of the element [45]. We found that tyrosine recombinase annotation with starfish largely overlapped with results from a separate blast search to identify DUF3435 homologues at the head of insertions. Overall, only a relatively small number of genes were in agreement as full *Starship* captains after downstream automated (starfish) or manual element inference (Fig. 5a). A gene tree of all tyrosine recombinase and putative captain genes showed the presence of three distinct lineages but no consistent clustering of either gene types or method of identifying them. Note the highly divergent nature of the genes and therefore the difficulty of alignment and subsequent poor branch support throughout the tree (Fig. 5b).

*Starship* size varied considerably, ranging from 34–688 kbp. *Gt*B strains harboured notably more elements, followed by *Ga* strains which included a nested element (Fig. 5c). *Gt*A and *Gh* strains each contained a single smaller (< 100 kbp) element, which in both cases we predict to have been vertically transmitted based on similar gene content and conserved location within the genome (Fig. 5a,c). *Gt*A elements were exceptional in that each was gene-poor and positive for element-wide RIP (average CRI = 0.2–0.3).

## Discussion

In this study we have established foundational genome resources for the genus *Gaeumannomyces*. A particular strength of the *Gt* assemblies reported here is the structural annotation methodology, which capitalised on the fact that five reference strains were sequenced, assembled and annotated in the same way, each with its own transcriptome but also employing a novel ‘multiple lift-off’ approach that provided additional evidence for robust gene models. Another benefit of the annotation approach is that the REAT-Mikado-minos pipeline [46] provides models for gene isoforms alongside the primary transcripts. Alternative splicing has been implicated in regulation of virulence in phytopathogens [47], for instance by mediating transcriptome remodelling during pathogenesis in *P. oryzae* [48]. Alternative splicing has also been reported to be more frequent in pathogens than non-pathogens [49], however we found a similar overall percentage of genes with multiple isoforms in *Gh* compared to *Gt* and *Ga* (Supplemental Fig. S2). There was perhaps a skew towards a greater proportion of genes with exactly two or three isoforms in *Gt*, particularly *Gt*A, raising the question as to whether this somehow relates to their apparent higher virulence in wheat. These rich annotation resources will allow further exploration of the isoform content of *Gaeumannomyces* and its potential role in virulence.

A major finding from our synteny analyses was the presence of a large chromosomal translocation in Gt-LH10 (Fig. 2). Similar largescale translocations have been identified in *Pyricularia* [50, 51]. It is entirely plausible that we have identified a genuine translocation, however confidence would be increased by obtaining Hi-C evidence and/or by corroborating with population-level data. It is also notable that this large translocation occurred in the same strain we found to have an expansion of TEs (Supplemental Fig. S6), as TEs have been found to mediate interchromosomal rearrangements [50, 52, 53]. Hi-C data would also allow us to robustly locate centromeres [54], which are also implicated in chromosomal rearrangements [55, 56]. Here we used a minimal approach to estimate potential centromeric regions, based simply on the fact that AT-rich regions are a common defining feature of centromeres in *P. oryzae* [57], which we also cross-checked with gene sparsity (Supplemental Fig. S3) — however, we were only able to distinguish potential centromeres for a subset of the pseudochromosomes.

In addition to the chromosomal translocation, Gt-LH10 also stood out from other strains in terms of TE content, with an expansion in total number of TEs (Supplemental Fig. S6) and smaller gene–TE distances (Fig. 3). Aside from the atypical features of the Gt-LH10 genome, there was additional intraspecific variability within the *Gt* A/B lineages in terms of both genome structure and gene content. For instance, there were strain-specific inversions (Fig. 2) and many of the HCN genes were present in low copy-number in one *Gt*A strain, but completely absent in the other (Fig. 4c). These findings emphasise the need for pangenome references, as an individual strain alone cannot sufficiently represent the variability across the whole species [58, 59]. The five *Gt* strains reported here can act as references for the UK, but future research must work towards building a global pangenome so that we can provide a reference for *Gt* which captures a fuller representation of the species.

Another structural feature that these high-quality assemblies allowed us to explore in *Gaeumannomyces* was genome compartmentalisation. A number of fungal phytopathogens exhibit TE- and effector-rich compartments that enable rapid evolution in the plant–fungal arms race, dubbed the ‘two-speed’ genome model [29], which has since been extended to ‘multi-speed’ models [60]. Accordingly, we hypothesised that we would find CSEPs and TEs to colocalise across our assemblies, however we did not find consistent evidence for such compartments in *Gaeumannomyces* (Fig. 3). Our results are not altogether surprising as a previous study of selection signatures in *Gt* and two other *Magnaporthales* taxa also found no evidence for multi-speed genomes [61]. We therefore consider *Gaeumannomyces* taxa to have ‘one-compartment’ genomes in relation to TE/effector content – a term that was introduced by Frantzeskakis et al. [60] for genomes that do not conform to the two- or multi-speed models, and with ‘compartment’ suggested as an alternative to ‘speed’ as the defining features of these compartments does not necessarily equate to them being fast-evolving [62]. With the rising number of high-quality genome resources, more examples are emerging that contradict the suggestion that phytopathogenicity is routinely accompanied by TE/effector compartmentalisation [60]. In fact, TE/effector compartmentalisation has been found in the non-pathogenic arbuscular mycorrhizal fungus *Rhizophagus irregularis* [63], and TE/virulence factor compartmentalisation has also been found in chytrid animal pathogens [64], demonstrating that it is not necessarily central to phytopathogenicity, but may instead be a mechanism driving genome plasticity in fungi of various lifestyles [62]. While we did not find compelling evidence for TE/effector compartmentalisation in *Gaeumannomyces*, we did observe non-random patterns in the distribution of CSEPs (Fig. 3a), which permutation analyses found to be closer to telomeric regions in a pseudochromosome-dependent manner (Supplemental Fig. S5b). This could suggest that alternative mechanisms of effector compartmentalisation may be at play.

Our results indicated conserved genetic machinery for plant cell wall deconstruction/modification across both pathogenic and non-pathogenic *Gaeumannomyces* (Fig. 4a, S11a), suggesting that the mechanism(s) by which species first colonise roots may be similar, if not the final outcome of the plant-fungal interaction [18]. Using spatial transcriptomics to visualise not only how *Gt* and *Gh* individually colonise wheat roots, but also how they interact with each other in the plant and the gene expression associated with this process, would undoubtedly shed light on this host–pathogen–antagonist system. Two putative orthologues of CSEP genes that have previously been implicated in pathogenicity were present in *Gt* and *Ga* pathogenic taxa but missing in non-pathogenic *Gh*, making them promising targets for future experiments to determine if either is important for *Gt* pathogenicity in wheat. *UvHrip1* (from *Ustilaginoidea virens*) is thought to be involved in suppressing host immunity and has already been reported in *Gt* [65], while *PBC1* (from *Pyrenopeziza brassicae*) is a cutinase implicated in host penetration [66]. It was intriguing that none of the CSEPs assigned to PHI-base genes were unique to *Gt*, perhaps suggesting that there is relatively high overlap in effector-mediated virulence mechanisms in *Gt* and *Ga*.

In a similar pattern to the CSEPs, BGCs were frequently scattered across the genus (Supplemental Fig. S10c). Although BGC variance was predominantly explained by relatedness (i.e. lineage or species) versus lifestyle (Fig. 4a), the discovery of two indole BGCs only present in *Gh* is intriguing as indole derivatives are known to mediate signalling between plants and fungi, and have been implicated in numerous mutualistic plant-fungal interactions [67–70]. We also found two BGCs with orthologues in clusters from other species which were present in *Gt* and *Ga* and absent in *Gh*. One was the *Fusarium heterosporum* equisetin BGC [71], an antibiotic and plant virulence factor in *Fusarium* spp. [72], for which there were orthologues for five out of a total eleven of the genes in the corresponding *Gaeumannomyces* cluster, largely rearranged (Supplemental Fig. S11e). The other *Gaeumannomyces* cluster missing in *Gh*, which had similarity to the *Aspergillus oryzae* dichlorodiaporthin BGC [73], contained orthologues for four out of six genes and in a more similar configuration (Supplemental Fig. S11d). However, the *Gaeumannomyces* clusters were missing an orthologue for the gene necessary for the chlorination of diaporthin in *A. oryzae* (aoiQ) [74], suggesting that these BGCs may be implicated in the production of other diaporthin derivatives than dichlorodiaporthin. Diaporthin and its derivatives have been reported as phytotoxins [75, 76], however they have also been reported from endophytic fungi with more broad antibacterial properties [77].

A BGC with similarity to the DHN melanin cluster of *Pestalotiopsis fici* [78] was present in all taxa, which is consistent with melanisation being characteristic of *Gaeumannomyces* species [79, 80]. However, while the clusters contained orthologues of the PfmaE PKS gene, which produces the melanin precursor 1,3,6,8-tetrahydroxynaphthalene (T4HN), they were missing orthologues for the PfmaG gene, a T4HN reductase which converts T4HN into the subsequent precursor scytalone, necessary for DHN melanin biosynthesis [81] (Supplemental Fig. S11a). As genes involved in DHN melanin biosynthesis are not always clustered together [82], a T4HN reductase necessary for scytalone production may be located elsewhere in the genome in *Gaeumannomyces* species. In *P. fici* itself, a putative second T4HN reductase (PfmaI) is indeed located outside the Pfma BGC [78]. Also present in all *Gaeumannomyces* genomes, including two copies in strain Gt-23d, was a BGC with similarity to the nectriapyrone BGC from *Pyricularia oryzae* [83], which included both the PKS (NEC1) and O-methyltransferase (NEC2) necessary for nectriapyrone biosynthesis, in the same arrangement (Supplemental Fig. S11b). Nectriapyrone is not implicated in plant pathogenicity in *P. oryzae*, but may be involved in interactions with other microbes [83]. A single oxidosqualene cyclase (occ) gene required for the biosynthesis of clavaric acid in *Hypholoma sublateritium* [84] also had orthologues in BGCs of all lineages, however the occ orthologue was missing from the type B strain Gt-4e (Supplemental Fig. S11c). Clavaric acid has been shown to have anticancer properties [85], but its role in the fungus is not known.

In terms of host range, *Gt* has been shown to have low avenacinase activity relative to *Ga* [13], which is understood to be the reason *Gt* is incapable of also infecting oat roots [86]. The avenacinase gene was nonetheless present in all strains across the genus; whether sequence polymorphism (Supplemental Fig. S8c) or differences in regulatory machinery are responsible for the variation in avenacinase activity remains to be determined. It is notable that *Gh* has also been found to be capable of colonising oat roots [17] despite greater divergence of the *Gh* avenacinase protein sequence from *Ga* when compared to *Gt* (Supplemental Fig. S8b).

In line with the common understanding that *Gt* is self-fertile or homothallic [2], we found both *MAT1-1* and *MAT1-2* idiomorphs to be present in the *GtA* and *GtB* strains. These idiomorphs were located on two unlinked MAT loci, an atypical but occasionally observed homothallic MAT locus architecture in ascomycetes [87–89]. Although it is homothallic, *Gt* is also capable of outcrossing [90, 91], the rates of which may be underestimated in many other homothallic fungi [92, 93]. Similarly to *Gt*, for *Ga* both MAT loci were identified. To our knowledge, the sex determination system of *Gh* has not previously been reported, but our results indicate only one idiomorph at a single MAT locus suggesting this species is self-sterile, or heterothallic. Evolutionary transitions between heterothallism and homothallism are common in ascomycetes [89, 94–96], but the implications on fitness are not fully understood. In the scenario of a fungus infecting a crop monoculture, it may be advantageous for the fungus to be homothallic when rapidly expanding across the niche, as it will not be delayed by a reliance on the presence of compatible mating types. A higher rate of outcrossing due to heterothallism could be unfavourable, as it could break up combinations that are already well adapted to the genetically uniform host [97]. However, there are also successful heterothallic crop pathogens (e.g. the most infamous *Magnaporthales* pathogen, *P. oryzae* [98]), demonstrating that there is no single best evolutionary strategy in this context.

An unanticipated result was the absence of HCN genes in the *Gt*A lineage (Fig. 4a), despite all other strains in the genus, including earlier diverging *Gh*, having genes which had undergone copy-number expansions (Supplemental Fig. S13). These HCN genes were on average significantly closer to TEs than other genes (Supplemental Fig. S12b), which aligns with the fact that TEs are known to play a role in gene duplication [99]. GO enrichment analysis identified a variety of fundamental biological processes to be significantly overrepresented amongst HCN genes in the other lineages: regulation of cellular pH and respiratory activity in non-pathogenic strains; and golgi organisation, protein localisation, mRNA cis-splicing and respiratory activity in pathogenic strains. As previously mentioned, alternative splicing has previously been linked to pathogenicity; respiratory activity has been shown to induce a developmental switch to symbiosis in an arbuscular mycorrhizal fungus [100]; and mediation of cellular pH by V-ATPase has specifically been linked to pathogenesis in *P. oryzae* [101], although here it was implicated in a non-pathogenic *Gh* strain. Further investigation into the specific function of these genes is required to determine whether any of these processes are essential to lifestyle or virulence in *Gaeumannomyces*. We should note that the total length of HCN genes was not sufficiently large to account for the overall greater genome size of *Gt*B compared to *Gt*A (Supplemental Table S1).

Gene duplicates are generally understood to be readily removed unless they serve to improve host fitness, for instance by favourably modifying expression levels or rendering a completely new function [102, 103]. RIP is a genome defence response against unchecked proliferation of duplicated sequences [104], which most frequently effects repetitive sequences, with the knock-on effect of reducing TE-mediated gene duplication, but also directly mutates duplicated coding regions [105]. In *Gaeumannomyces* we found 10–14% of the genome contained signatures of RIP, which is a moderate level relative to other ascomycetes, e.g. *Pyronema confluens* (0.5%) [106], *Fusarium* spp. (< 1–6%) [107], *Neurospora* spp. (8–23%) [108], *Zymoseptoria tritici* (14–35%) [109] and *Hymenoscyphus* spp. (24–41%) [110]. Genome-wide RIP was highest in *Gt*A, which was consistent with its low level of gene duplication (e.g. [111]), but not fully explanatory as *Gh* had only marginally lower levels of RIP while still maintaining HCN outliers. We can only presume that *Gt*A strains have been under stronger selective pressures to remove duplicates, although the evolutionary mechanisms driving this requires further investigation.

There was a similar pattern when exploring the RIP patterns across giant transposable *Starship* elements. We found only a single *Starship* in *Gt*A strains, which was gene-poor and had undergone extensive RIP (Fig. 5b), supporting the idea that this lineage employs stringent genome defence measures. By contrast, *Gt*B strains contained a proliferation of *Starships*, including one closely approaching the largest size reported thus far [112]. We expect that the increased availability of highly contiguous, long-read assemblies such as we report here will make the upper size extremes of such giant TEs more feasible to detect [113]. Giant cargo-carrying TEs that can be both vertically and horizontally transmitted were first identified in bacteria [114]. Recently the *Starship* superfamily was identified as specific to and widespread in the *Pezizomycotina* subphylum and, aside from the characteristic ‘captain’ tyrosine recombinase gene, each *Starship* contains a highly variable cargo [25]. Mobilisation of cargo genes by *Starships* has been linked to the acquisition of various adaptive traits in fungal species, such as metal resistance [115], formaldehyde resistance [112], virulence [116], climatic adaptation [117] and lifestyle switching [25]. However, *Starships* are not inherently beneficial to the fungal host. One of the earliest groups of genes associated with the cargo of certain *Starships* was spore-killer or Spok genes, which bias their own transmission via the process of meiotic drive (i.e. by killing spores that do not inherit them) [118]. By incorporating Spok genes, a *Starship* element also biases its transmission, leading to it being referred to as a ‘genomic hyperparasite’ [119]. This corresponds to the concept of TEs as selfish genetic elements, which can prevail in the genome despite being neutral or deleterious to the overall fitness of the host. Whether mobilisation of an element and associated cargo is beneficial or detrimental to the host, TEs such as *Starships* are nonetheless drivers of genome evolution. Further detailed investigation of the specific cargo in the elements we have identified in *Gaeumannomyces* is a priority to explore how these giant TEs may be contributing to lifestyle and virulence.

While the differences in the overall appearance of the wheat plants and their root systems when infected with *Gt*A versus *Gt*B were visually compelling (Fig. 1A), our sample size was extremely limited and the quantitative data did not show such a strong distinction (Fig. 1C). A study by Lebreton et al. [11] with a much larger sample size found *Gt* type A strains to be significantly more aggressive in vitro despite high intraspecific variability in take-all severity (type A = G2 in their study [8]). The dominance of type A strains in a site has also been reported to positively correlate with disease severity [12]. It is also notable that five out of six wheat plants which died were inoculated with *Gt*A strains. Our phylogenomic analysis confirmed with significant branch support that the two lineages are indeed monophyletic (Supplemental Fig. S14b) and, together with our comparative genomics results, the question naturally arises as to whether *Gt*A and *Gt*B are in fact distinct species. If we compare *Ga* and *Gt* in terms of synteny, genome size and gene content, the magnitude of differences does not appear to be more pronounced than those between *Gt*A and *Gt*B. Host alone is not a sufficient distinction since, despite being a separate species, *Ga* is also able to infect wheat, although rarely found to do so [7]. Lebreton et al. [11] suggested that ‘genetic exchanges between [A and B] groups are rare events or even do not exist’, but this was based on analysis of a limited number of genetic markers. Much broader whole-genome sequencing efforts are required to assess gene flow between lineages at the population-level, as well as the level of recombination. Understanding population dynamics could also shed light on the observed changes in ratio of *Gt*A and *Gt*B across wheat cropping years [11], which has implications for strategic crop protection measures.

## Conclusions

We have generated near-complete assemblies with robust annotations for under-explored but agriculturally important wheat-associated *Gaeumannomyces* species. In doing so we confirmed that *Gaeumannomyces* taxa have one-compartment genomes in the context of TE/effector colocalisation, however the presence of giant cargo-carrying *Starship* TEs may contribute to genomic plasticity. Genomic signatures support the separation of *Gt* into two distinct lineages, with copy-number as a potential mechanism underlying differences in virulence. Regarding differences between pathogenic *Gt* and non-pathogenic *Gh*, we found that *Gh* has a larger overall genome size and greater number of genes. We also identified a number of BGCs present in *Gt* and *Ga* but absent in *Gh* and vice versa, including two indoles and equisetin-like and dichlorodiaporthin-like BGCs, which may be key factors contributing to lifestyle differences. In addition to providing foundational data to better understand this host–pathogen–antagonist system, these new resources are also an important step towards developing much-needed molecular diagnostics for take-all, whether conventional amplicon sequencing, rapid in situ assays [120] or whole-genome/metagenomic sequencing approaches [121]. Future research will require whole-genome sequencing of taxa from a broader geographical range to produce a global pangenome, which will provide a comprehensive reference for expression analyses to explore the role of virulence in *Gt* lineages, as well as population genomics to shed light on their evolution and distribution.

## Methods

### Samples

Nine *Gaeumannomyces* strains were selected from the Rothamsted Research culture collections, including five *Gt* strains (two type A and three type B), two *Ga* strains and two *Gh* strains (Supplemental Table S2). All were collected from various experimental fields at Rothamsted Farm [122] between 2014 and 2018.

### *G. tritici* virulence test in adult wheat plants

To test the virulence of the five *Gt* strains, we performed inoculations of each strain (six replicates) into the highly susceptible winter wheat cultivar Hereward. First the roots of seedling plants were inoculated with the fungus by using plastic drinking cups (7.5 cm wide × 11 cm tall) as pots, ensuring that all seedlings were well colonised before transferring to a larger pot. Pots were drilled with four drainage holes 3 mm in diameter. A 50 cm^3^ layer of damp sand was added to each pot, followed by a 275 g layer of naïve soil collected from a field at Rothamsted Farm after a non-legume break crop. Inoculum was prepared by taking a 9 mm fungal plug with a cork borer number 6 from the outer part of a fungal colony grown on a potato dextrose agar (PDA) plate and mixing with sand to make up a 25 g inoculum layer. A final 150 g layer of naïve soil was added on top of the inoculum layer. One wheat seed was sown on the surface of the soil and covered with a 50 cm^3^ layer of grit to aid germination and create a humid environment for fungal colonisation. Pots were watered well and placed in a controlled environment room (16 h day, light intensity 250 μmols, 15°C day, 10°C night, watered twice a week from above). A randomised block design was generated in Genstat 20th Edition to take potential environmental differences across the growth room into account.

After two weeks of growth, each wheat seedling in a small pot was transferred by removing the plastic cup and placing the entire contents undisturbed into a larger 20 cm diameter pot containing a 2 cm layer of clay drainage pebbles. Three small pots were transferred to each large pot and filled in with more soil, resulting in three plants per pot. There were 6 replicates for each treatment, and a control pot with no fungus was also set up in the same manner, but a PDA plate without fungus was used for preparing the inoculum layer. The pots were transferred to a screenhouse and arranged randomly within blocks containing one pot per treatment. The pots were established in September and remained outside in the screenhouse to ensure exposure to winter conditions and therefore allow plant vernalisation to take place.

Measurements of the above-ground characteristics were first undertaken to note the severity of any take-all symptoms once the floral spike (ear) was fully emerged. The height of each labelled plant was measured from the stem base to the tip of the ear to the nearest 0.5 cm to identify whether there was stunted growth. Additionally, the length of the ear and flag leaf were recorded, again to the nearest 0.5 cm. The number of ears per plant was also recorded.

For below-ground measurements, the pots were washed out post full plant senescence and the plants were well rinsed to remove the soil while minimising damage to the roots. Any roots that broke off were collected and put into the cup with the main plants to maintain accuracy of the biomass measurements. The stems were then cut about 10 cm from the base. The plants were placed in a white tray filled with water to enable clear observation of the roots. The number of tillers for each plant was counted. The severity of take-all infection was then estimated by using the Take-All Index (TAI), classified through the following categories: Slight 1 (0–10% of roots infected), slight 2 (11–25%), moderate 1 (25–50%), moderate 2 (51–75%) and severe (76–100%). This was then input into the following formula: TAI = ((1 x % plants slight 1) + (2 x % plants slight 2) + (3 x % plants moderate 1) + (4 x % plants moderate 2) + (5 x % plants severe)) / 5 [26]. Following this, the length of the roots was measured to the nearest 0.5 cm. By cutting off one root at a time, the number of roots for each plant was counted and the roots transferred into cardboard trays, one per pot. These were then dried at 80°C on metal trays for 16 h. One tray at a time was removed from the oven to reduce any moisture gain before weighing. The dried root biomass per pot was then recorded.

To statistically test for mean differences in the various characteristics between strains, we first made Q-Q plots using the ggqqplot function from ggpubr v0.6.0 [123] to confirm approximate data normality. We then used the levene_test function from the package rstatix v0.7.2 [124] to assess the assumption of homogeneity of variance, where a significant p value (p < 0.05) means that the assumption is violated. If we could ascertain homogeneity of variance, a multiple comparison test between strains was performed with the tukey_hsd rstatix function. Where homogeneity of variance was violated, the games_howell_test rstatix function was instead used for multiple comparison testing [125].

### Genome sequencing

For DNA and RNA extractions of all nine *Gaeumannomyces* taxa, a 4 mm plug of mycelium from axenic cultures was transferred to 500 ml of potato dextrose broth treated with penicillium/streptomycin (10,000 U/mL) using a sterile 4 mm corer. Cultures were grown at 20°C in dark conditions on an orbital shaker at 140 rpm for ~ 7–14 days. Mycelia were collected via vacuum filtration and flash frozen using liquid nitrogen and stored at −80°C, before grinding with a sterilised mortar and pestle until a fine powder was created.

DNA was extracted using one of two kits: the Phytopure Nucleon Genomic DNA kit (Cytiva, MA, USA) eluted in 50 µl low-pH TE buffer; and the NucleoBond HMW DNA kit (Macherey–Nagel, North Rhine- Westphalia, Germany) eluted in 100 µl–200 µl low-pH TE buffer. The manufacturer’s protocols were modified to optimise for high molecular weight [126]. Sufficient DNA concentration (50 ng/µl DNA) was confirmed by Qubit fluorometer (Invitrogen, MA, USA) and purity (260/280 absorbance ratio of approximately 1.6–2.0 and 260/230 absorbance ratio of approximately 1.8–2.4) confirmed with a NanoDrop spectrophotometer (Thermo Fisher Scientific, MA, USA). Sufficient strand lengths (80% > 40 Kbp length) were confirmed using the Femto Pulse System (Agilent Technologies, Inc, CA, USA).

RNA from the same sample material was extracted using the Quick-RNA Fungal/Bacterial miniprep kit (Zymo Research, CA, USA) using the manufacturer’s protocol and eluted in 25 µl of DNase/RNase free water. Sufficient RNA concentration (71 ng/µl RNA) was confirmed by Qubit fluorometer (Invitrogen, MA, USA) and purity (260/280 absorbance ratio of approximately 1.8–2.1 and 260/230 absorbance ratio of > 2.0) confirmed with a NanoDrop spectrophotometer (Thermo Fisher Scientific, MA, USA). An RNA integrity number > 8 was confirmed by Bioanalyzer RNA analysis (Agilent Technologies, Inc, CA, USA).

DNA and RNA extractions were sent to the Genomics Pipelines Group (Earlham Institute, Norwich, UK) for library preparation and sequencing. 2–5.5 µg of each sample was sheared using the Megaruptor 3 instrument (Diagenode, Liege, Belgium) at 18-20ng/µl and speed setting 31. Each sample underwent AMPure PB bead (PacBio, CA, USA) purification and concentration before undergoing library preparation using the SMRTbell Express Template Prep Kit 2.0 (PacBio) and barcoded using barcoded overhang adapters 8A/B (PacBio) and nuclease treated with SMRTbell enzyme cleanup kit 1.0 (PacBio). The resulting libraries were quantified by fluorescence (Invitrogen Qubit 3.0) and library size was estimated from a smear analysis performed on the Femto Pulse System (Agilent). The libraries were equimolar pooled into four multiplex pools and each pool was size fractionated using the SageELF system (Sage Science, MA, USA), 0.75% cassette (Sage Science). The resulting fractions were quantified by fluorescence via Qubit and size estimated from a smear analysis performed on the Femto Pulse System, and 1–2 fractions per pool were selected for sequencing and pooled equimolar to have equal representation of barcodes per pool. The loading calculations for sequencing were completed using the PacBio SMRTLink Binding Calculator v10.1.0.119528 or v10.2.0.133424. Sequencing primer v2 or v5 was annealed to the adapter sequence of the library pools. Binding of the library pools to the sequencing polymerase was completed using Sequel II Binding Kit v2.0 or 2.2 (PacBio). Calculations for primer to template and polymerase to template binding ratios were kept at default values. Sequel II DNA internal control was spiked into the library pool complexes at the standard concentration prior to sequencing. The sequencing chemistry used was Sequel II Sequencing Plate 2.0 (PacBio) and the Instrument Control Software v10.1.0.119549 or 10.1.0.125432. Each pool was sequenced on 1–2 Sequel II SMRTcells 8M (PacBio) on the Sequel IIe instrument. The parameters for sequencing were as follows: CCS sequencing mode; 30-h movie; 2-h adaptive loading set to 0.85 or diffusion loading; 2-h immobilisation time; 2–4-h pre-extension time; and 70–86pM on plate loading concentration.

RNA libraries were constructed using the NEBNext Ultra II RNA Library prep for Illumina kit (New England Biolabs, MA, USA), NEBNext Poly(A) mRNA Magnetic Isolation Module and NEBNext Multiplex Oligos for Illumina (96 Unique Dual Index Primer Pairs) at a concentration of 10 µM. RNA libraries were equimolar pooled, q-PCR was performed, and the pool was sequenced on the Illumina NovaSeq 6000 (Illumina, CA, USA) on one lane of a NVS300S4 flowcell with v1.5 chemistry producing a total of 3,370,873,981 reads.

### Genome assembly

See Supplemental Fig. S1a for a schematic summarising the bioinformatics workflow. HiFi reads were assembled using hifiasm v0.16.1-r375 [127] with the -l 0 option to disable purging of duplicates in these haploid assemblies. The assemblies were checked for content correctness with respect to the input HiFi reads using the COMP tool from KAT v2.3.4 [128], and QUAST v5.0.2 [129] was used to calculate contiguity statistics. BlobTools v1.0.1 [130] was used to check for contamination (Supplemental Fig. S15) — this required a hits file, which we produced by searching contigs against the nt database (downloaded 21/05/2021) using blastn v2.10, and a BAM file of mapped HiFi reads, which we produced using minimap2 v2.21 [131] and samtools v1.13 [132].

Gene set completeness was assessed using the ascomycota_odb10.2020–09–10 dataset in BUSCO v5.2.1 [133]. This revealed some gene duplication due to the presence of small contigs that had exceptionally low coverage (median of 1 across each small sequence) when projecting the kmer spectra of the reads onto them using KAT’s SECT tool. This was taken as evidence that the sequences did not belong in the assemblies. A custom script was written to filter out these small, low-coverage sequences, using the output of KAT SECT. KAT COMP, BUSCO and QUAST were re-run for the coverage filtered assemblies to verify that duplicated genes were removed without losing core gene content and produce final assembly contiguity statistics (Supplemental Fig. S16, Supplemental Table S1).

### Genome annotation

Repeats were identified and masked using RepeatModeler v1.0.11 [134] and RepeatMasker v4.0.7 [135] via EIRepeat v1.1.0 [136]. Gene models were annotated via the Robust and Extendable Eukaryotic Annotation Toolkit (REAT) v0.6.3 [46] and MINOS v1.9 [137]. The REAT workflow consists of three submodules: transcriptome, homology, and prediction. The transcriptome module utilised Illumina RNA-Seq data, reads that were mapped to the genome with HISAT2 v2.1.0 [138] and high-confidence splice junctions identified by Portcullis v1.2.4 [139]. The aligned reads were assembled for each tissue with StringTie2 v1.3.3 [140] and Scallop v0.10.2 [141]. A filtered set of non-redundant gene models were derived from the combined set of RNA-Seq assemblies using Mikado v2.3.4 [142]. The REAT homology workflow was used to generate gene models based on alignment of protein sequences from publicly available annotations of 27 related species (Supplemental Table S3) and a set of proteins downloaded from UniProt including all the proteins from the class *Sordariomycetes* (taxid:147,550) and excluding all proteins from the publicly available annotation of *Gt* R3-111a-1 (GCF_000145635). The prediction module generated evidence-guided models based on transcriptome and proteins alignments using AUGUSTUS v3.4.0 [143], with four alternative configurations and weightings of evidence, and EVidenceModeler v1.1.1 [144]. In addition, gene models from the *Gt* R3-111a-1 annotation were projected via Liftoff v1.5.1 [145], and filtered via the multicompare script from the ei-liftover pipeline [146], ensuring only models with consistent gene structures between the original and transferred models were retained.

The filtered Liftoff, REAT transcriptome, homology and prediction gene models were used in MINOS to generate a consolidated gene set with models selected based on evidence support and their intrinsic features. Confidence and biotype classification was determined for all gene models based on available evidence, such as homology support and expression. TE gene classification was based on overlap with identified repeats (> 40 bp repeat overlap).

To make best use of having multiple identically generated annotations for the genus, we opted to additionally repeat a lift-over process projecting the gene models from each MINOS run to all nine assemblies. We then removed gene models overlapping rRNA genes from the multiple-lift-over annotations and the previously consolidated MINOS annotation using RNAmmer v1.2 [147] and BEDTools v2.28 [148]. The MINOS consolidation stage was repeated using four files as input: the high-confidence models from the lift-over; the high-confidence genes of the previous MINOS run for the specific assembly; the low-confidence models of the previous MINOS run for the specific assembly; and the low-confidence models of the lift-over of all the closely related species. This multiple-lift-over approach allowed us to cross-check gene sets across strains and determine whether missing genes were truly absent from individual assemblies or had just been missed by the annotation process. Finally, mitochondrial contigs were identified using the MitoHiFi v2.14.2 pipeline [149], with gene annotation using MitoFinder v1.4.1 [150] and the mitochondrion sequence from *Epichloë novae-zelandiae* AL0725 as a reference (GenBank accession NC_072722.1).

Functional annotation of the gene models was performed using AHRD v3.3.3 [151], with evidence from blastp v2.6.0 searches against the Swiss-Prot and TrEMBL databases (both downloaded on 19/10/2022), and mapping of domain names using InterProScan v5.22.61 [152]. Additional annotations were produced using eggNOG-mapper v2.1.9 [153] with sequence searches against the eggNOG orthology database [154] using DIAMOND v2.0.9 [155]. CAZymes were predicted using run_dbcan v3.0.1 [156] from the dbCAN2 CAZyme annotation server [157] this process involved (i) HMMER v3.3.2 [158] search against the dbCAN HMM (hidden Markov model) database; (ii) DIAMOND v2.0.14 search against the CAZy pre-annotated CAZyme sequence database [159] and (iii) eCAMI [160] search against a CAZyme short peptide library for classification and motif identification. A gene was classified as a CAZyme if all three methods were in agreement.

CSEPs were predicted using a similar approach to Hill et al. [161], with some additions/substitutions of tools informed by Jones et al. [162]; see Supplemental Fig. S1b for a schematic overview. Briefly, we integrated evidence from SignalP v3.0 [163], v4.1g [164], v6.0g [165]; TargetP v2.0 [166]; DeepSig v1.2.5 [167]; Phobius v1.01 [168]; TMHMM v2.0c [169]; Deeploc v1.0 [170]; ps_scan v1.86 [171]; and EffectorP v1.0 [39], v2.0 [40] and v3.0 [41]. CSEPs were then matched to experimentally verified genes in the PHI-base database [38] (downloaded 21/07/2023) using a BLAST v2.10 blastp search with an e-value cutoff of 1e-25. In the event of multiple successful hits, the hit with the top bitscore was used. Secondary metabolites were predicted using antiSMASH v7.1.0 [172]. Reference protein sequences for avenacinase from *Ga* (GenBank accession AAB09777.1) and mating-type locus idiomorphs *MAT1-1* and *MAT1-2* from *Pyricularia grisea* [173] were used to identify their respective genes in each of the nine assemblies using a blastp search (e-value cutoff 1e-25).

### Phylogenetic classification of *G. tritici* types

To confirm the classification of *Gt* strains within established genetic groups — sensu Daval et al. [8] and Freeman et al. [9] — gene trees were produced for gentisate 1,2-dioxygenase (*gdo*; GenBank accessions FJ717712–FJ717728) and *ITS2*. GenePull [174] was used to extract the two marker sequences from the new assemblies reported here. *ITS2* could not be found in the existing *Gt* R3-111a-1 assembly (RefSeq accession GCF_000145635.1), so that strain was only included in the *gdo* gene tree. We aligned each marker gene separately using MAFFT v7.271 [175] and manually checked the gene alignments. The gene trees were estimated using RAxML-NG v1.1.0 [176] and the GTR + G nucleotide substitution model (Supplemental Fig. S14a). Branch support was computed using 1,000 Felsenstein’s bootstrap replicates, or until convergence according to the default 3% cutoff for weighted Robinson-Foulds distances [177], whichever occurred first. An avenacinase gene tree was produced in the same way but using the JTT + G4 amino acid substitution model.

### Phylogenomics of *Gaeumannomyces*

A genome-scale species tree was produced to provide evolutionary context for comparative analyses. We used OrthoFinder v2.5.4 [178] to cluster predicted gene models for primary transcripts into orthogroups — in addition to the newly sequenced *Gaeumannomyces* taxa, this also included *Gt* R3-111a-1 and the outgroup *Magnaporthiopsis poae* ATCC 64411 (GenBank accession GCA_000193285.1). Alongside the coalescent species tree produced within OrthoFinder by STAG [179], we also used a concatenation-based approach. We used MAFFT to produce gene alignments for 7,029 single-copy phylogenetic hierarchical orthogroups or HOGs (hereafter, genes) that were present in all taxa. These were trimmed using trimAl v1.4.rev15 [180], concatenated using AMAS [181] and run in RAxML-NG [176] with genes partitioned and the JTT + G4 amino acid substitution model. Branch support was calculated as above.

Alongside the species tree we visualised assembly N50; the number of gene models; the proportion of these that were functionally annotated by AHRD; and the number of unassigned gene models from OrthoFinder (Supplemental Fig. S17). Due to concerns regarding the comparability of the existing *Gt* R3-111a-1 annotation to the strains reported in this study, and to avoid introducing computational bias, the existing *Gt* R3-111a-1 annotation was excluded from downstream comparative analyses for the sake of consistency.

### Genome structure and synteny

To identify both potential misassemblies and real structural novelty in our strains, we used GENESPACE v1.1.8 [28] to visualise syntenic blocks across the genomes. Fragments were considered to have telomeres at the ends if Tapestry v1.0.0 [182] identified at least five telomeric repeats (TTAGGG), and this was used together with the GENESPACE results to inform pseudochromosome designation. Telomeric repeats were also cross-checked with results from tidk v0.2.31 [183]. We calculated GC content across pseudochromosomes in 100,000 bp windows using BEDTools v2.29.2 [148], and TE, gene and CSEP density were calculated in 100,000 bp windows with a custom script, plot_ideograms.R. The composite RIP index (CRI) [43] was calculated in 500 bp windows using RIP_index_calculation.pl [184].

To statistically test for correlations between CSEP density and TE and /or gene density, we again made Q-Q plots using the ggqqplot function and the shapiro.test function to assess approximate data normality. This being violated, we calculated Kendall’s tau for each strain (rstatix cor_test function, method = "kendall"). The assumption of normality being similarly violated for distances from CSEPs/other genes to the closest TE, we performed a Wilcoxon rank sum test (wilcox_test function) to compare mean distances for CSEPs versus other genes for each strain. To compare the mean gene–TE distance across strains, we used a Games-Howell test (games_howell_test function) for multiple comparison testing. Comparison of distances between HCN genes and TEs versus other genes and TEs was tested in the same way.

We also performed permutation tests of CSEP–TE distances using the meanDistance evaluation function from the R package regioneR v1.32.0 [185], with the resampleRegions function used for randomisation of the gene universe over 1,000 permutations. Permutation tests of CSEP–telomere distances were performed in the same way, having assigned the first and last 10,000 bp of each pseudochromosome as telomeric regions.

### Comparative genomics

Functional annotations were mapped to orthogroups using a custom script, orthogroup_assigner.R, adapted from Hill et al. [161], which also involved retrieval of CAZyme names from the ExplorEnz website [186] using the package rvest v1.0.3 [187]. CAZyme families known to act on the major plant cell wall substrates were classified as by Hill et al. [161] based on the literature [188–193]. For *Gt*, gene content was categorised as core (present in all strains), accessory (present in at least two strains) and specific (present in one strain).

Broadscale differences in gene repertoires due to lifestyle (pathogenic *Gt* and *Ga* and non-pathogenic *Gh*) were statistically tested using a permutational analysis of variance (PERMANOVA) approach to estimate residual variance of gene content after accounting for variance explained by phylogenetic distance [30]. To analyse the potential for secondary metabolite production with this PERMANOVA approach, a presence-absence matrix for biosynthetic gene cluster families was produced from the antiSMASH results using BiG-SCAPE v1.1.5 [194], which additionally compared resulting clusters to known BGCs in the MIBiG repository [42] that were visualised using clinker v0.0.31 [195].

Gene duplicates were categorised as intrachromosomal (on the same pseudochromosome) or interchromosomal (on a different pseudochromosome) using the pangenes output files from GENESPACE. We conducted gene ontology (GO) enrichment analysis for high copy-number (HCN) genes using the R package topGO v2.50.0 [196] with Fisher’s exact test and the weight01 algorithm.

### *Starship* element identification

Giant transposable *Starship* elements were identified in our assemblies after noting dense blocks of transposons forming gaps between annotated genes. Manual inspection of these regions via synteny plots built with OMA v2.5.0 [197] and Circos v0.69 [198] revealed *Starship*-sized insertions [25], and an NCBI blastp search of the first gene in one such insertion in strain Gt-8d (Gt-8d_EIv1_0041140) returned 85% identity with an established *Gt* R3-111a-1 DUF3435 gene (GenBank accession EJT80010.1). These two genes were then used for a local blastp v2.13.0 search against all nine *Gaeumannomyces* assemblies reported here, which identified 33 full length hits (> 95% identity) that were associated with insertions when visualised in Circos plots. This manual approach was then compared to *Starship* element identification using starfish v1.0 [44]. Out of the total 28 elements predicted by starfish, 8 were flagged as potential false positives upon manual inspection. One element identified by starfish was discounted as it consisted solely of a single predicted captain gene with no cargo or flanking repeats. A gene tree of all tyrosine recombinases predicted by starfish (including *Starship* captains), blastp-identified DUF3435 homologues, and previously reported *Starship* captain genes [25] was built using the same methods described above for phylogenetic classification and the JTT + G4 amino acid substitution model, with the addition of alignment trimming using trimAl v1.4.rev15 [180] with the -gappyout parameter.

Data visualisation was completed in R v4.3.1 [199] using the packages ape v5.7–1 [200], aplot v0.2.2 [201], ComplexUpset v1.3.3 [202], cowplot v1.1.1 [203], data.table v1.14.8 [204], eulerr v7.0.0 [205], ggforce v0.4.1 [206], ggh4x v0.2.6 [207], gggenomes v0.9.12.9000 [208], ggmsa v1.6.0 [209], ggnewscale v0.4.9 [210], ggplot2 v3.4.4 [211], ggplotify v0.1.2 [212], ggpubr v0.6.0 [123], ggrepel v0.9.3 [213], ggtree v3.9.1 [214], Gviz v1.44.2 [215], matrixStats v1.0.0 [216], multcompView v0.1–9 [217], patchwork v1.1.3 [218], rtracklayer v1.60.1 [219], scales v1.2.1 [220], seqmagick v0.1.6 [221], tidyverse v2.0.0 [222]. All analysis scripts are available at https://github.com/Rowena-h/GaeumannomycesGenomics.

## Supplementary Information

Supplementary Material 1

## Data Availability

WGS data and annotated genome assemblies are available on GenBank under the BioProject accession PRJNA935249, or alternatively are deposited in Zenodo doi: 10.5281/zenodo.14823851 along with additional data files. All bioinformatics scripts are available at https://github.com/Rowena-h/GaeumannomycesGenomics.
