# Supplementary material for: Evolutionary genomics reveals variation in structure and genetic content implicated in virulence and lifestyle in the genus Gaeumannomyces
Source: BMC Genomics. 2025 Mar 12;26:239. doi: 10.1186/s12864-025-11432-0 (PMC11905480; doi:10.1186/s12864-025-11432-0)
Supplement: Supplementary file 1 — Supplementary Material 1 [file 12864_2025_11432_MOESM1_ESM.pdf]

SUPPLEMENTARY MATERIAL

Evolutionary genomics reveals variation in structure and genetic content implicated in virulence and lifestyle in the genus *Gaeumannomyces*

Rowena Hill[1], Michelle Grey[1], Mariano Olivera Fedi[1], Daniel Smith[2], Sabrina J. Ward[1], Gail Canning[2], Naomi Irish[1], Jade Smith[2], Vanessa E. McMillan[3], Jess Hammond[2], Sarah-Jane Osborne[2,4], Gillian Reynolds[1], Ellie Smith[1], Tania Chancellor[2,5], David Swarbreck[1], Neil Hall[1,6], Javier Palma-Guerrero[2,7], Kim E. Hammond-Kosack[2], Mark McMullan[1]

[1] Earlham Institute, Norwich Research Park, Norwich, NR4 7UZ, UK

[2] Rothamsted Research, Harpenden, AL5 2JQ, UK

[3] NIAB, 93 Lawrence Weaver Road, Cambridge, CB3 0LE, UK

[4] AHDB, Middlemarch Business Park, Siskin Parkway East, Coventry, CV3 4PE, UK

[5] Crop Science Centre, Department of Plant Sciences, University of Cambridge, 93 Lawrence Weaver Road, Cambridge, CB3 0LE, UK

[6] School of Biological Sciences, University of East Anglia, Norwich, NR4 7TJ, UK

[7] Research Institute of Organic Agriculture FiBL, Frick, 5070, Switzerland.

Table S1 Statistics for the new assemblies reported in this study. T-T = telomere-to-telomere contigs.

		G avenae		***G. hyphopodioides***		***G. tritici* type A**		***G. tritici* type B**		
		Ga-CB1	Ga-3aA1	Gh-1B17	Gh-2C17	Gt-8d	Gt-19d1	Gt-23d	Gt-4e	Gt-LH10
Nuclear genome	# contigs [≥1 Mbp;T-T]	37 [9;2]	62 [8;1]	69 [8;2]	49 [7;4]	41 [8;4]	63 [7;3]	54 [7;2]	45 [6;4]	332 [6;3]
	Largest contig (bp)	7,079,356	9,242,662	8,478,668	8,472,645	9,558,045	8,750,995	9,793,078	10,701,577	15,505,531
	Total size (bp)	41,409,597	41,681,452	48,861,344	45,138,018	41,248,712	41,352,696	44,534,174	43,603,089	52,080,895
	GC (%)	56.93	56.77	57.25	57.46	56.54	56.35	57.05	57.31	54.08
	N50	4,890,083	6,143,224	6,286,456	6,996,108	6,032,443	6,009,945	6,017,529	6,461,149	5,872,440
	Total size of chromosomes (bp)	40,127,652	39,513,041	44,435,645	43,779,550	40,223,851	39,569,350	42,655,128	42,095,333	42,729,530
	Completeness (single-copy BUSCOs)	1,654 (97.0%)	1,654 (97.0%)	1,587 (93.0%)	1,656 (97.1%)	1,652 (96.8%)	1,664 (97.5%)	1,656 (97.1%)	1,662 (97.4%)	1,662 (97.4%)
	# gene models	11,838	11,802	13,436	12,672	11,788	11,666	13,101	13,009	12,933
	# high confidence/low confidence protein-coding gene models	9,958/1,549	9,964/1,481	11,311/1,737	10,771/1,567	9,959/1,581	9,909/1,507	10,509/1,979	10,370/2,005	10,414/1,980
	Genome-wide RIP (%)	12.44	12.41	13.62	13.50	13.66	14.00	10.60	10.82	11.08
Mitogenome	Mitogenome size (bp)	167,275	172,445	113,602	116,357	174,693	174,693	190,005	182,019	60,839
	GC (%)	31.41	31.57	30.7	30.72	31.59	31.59	31.42	31.42	31.17
	# gene models	39	39	40	40	38	38	38	38	23

Table S2 Metadata for the *Gaeumannomyces* strains sequenced in this study. *Soil cores collected post-grain harvest of a wheat plot.

Species	Strain	Host/substrate	Collection year	Rothamsted Research field
Gaeumannomyces avenae	Ga-CB1	Common bent (*Agrostis capillaris*)	2018	Park Grass
G. avenae	Ga-3aA1	Soil	2018	Park Grass
G. hyphopodioides	Gh-1B17	Wheat (*Triticum aestivum*, Hereward)	2016	New Zealand
G. hyphopodioides	Gh-2C17	Wheat (*Triticum aestivum*, Scout)	2016	New Zealand
G. tritici	Gt-8d	Soil*	2017	Long Hoos
G. tritici	Gt-19d1	Soil*	2017	Long Hoos
G. tritici	Gt-23d	Soil*	2017	Long Hoos
G. tritici	Gt-4e	Soil*	2017	Long Hoos
G. tritici	Gt-LH10	Soil*	2014	Long Hoos

Table S3 NCBI accessions for assemblies which were used to inform structural annotation.

Strain	Accession
Magnaporthiopsis poae ATCC 64411	GCA_000193285
Valsa mali 03-8	GCA_000818155
Epichloë festucae Fl1	GCA_003814445
Pyricularia sp. CBS 133598	GCA_004337975
Fusarium culmorum Class2-1B	GCA_016952355
Trichoderma simmonsii GH-Sj1	GCA_019565615
Trichoderma semiorbis FJ059	GCA_020045945
Trichoderma cornu-damae KA19-0412C	GCA_020631695
Fusarium solani-melongenae CRI 24-3	GCA_023101225
Colletotrichum lupini IMI 504893	GCA_023278565
Pyricularia oryzae 70-15	GCF_000002495
Neurospora crassa OR74A	GCF_000182925
Fusarium verticillioides 7600	GCF_000149555
Thermothelomyces thermophilus ATCC 42464	GCF_000226095
Thermothielavioides terrestris NRRL 8126	GCF_000226115
Fusarium graminearum PH-1	GCF_000240135
Fusarium pseudograminearum CS3096 FP7	GCF_000303195
Ustilaginoidea virens UV-8b	GCF_000687475
Drechmeria coniospora ARSEF 6962	GCF_001625195
Pochonia chlamydosporia 170	GCF_001653235
Colletotrichum higginsianum IMI 349063	GCF_001672515
Pyricularia pennisetigena PpBr36	GCF_004337985
Pyricularia grisea PgNI	GCF_004355905
Fusarium poae DAOMC 252244	GCF_019609905
Fusarium musae F31	GCF_019915245
Purpureocillium takamizusanense PT3	GCF_022605165
Fusarium venenatum A3/5	GCF_900007375

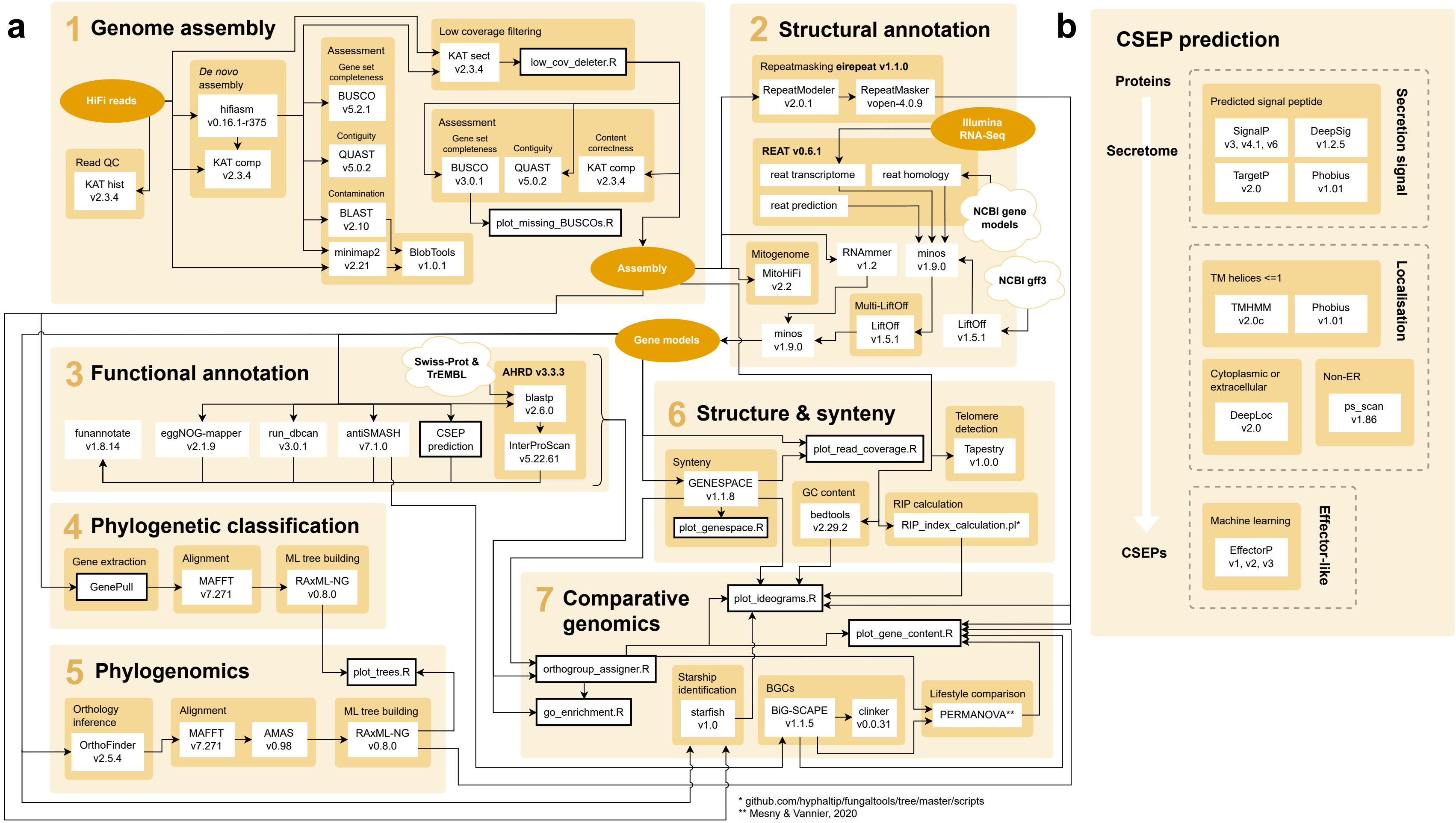

Fig. S1 **a** Schematic summarising the bioinformatics analysis workflow developed in this study, available at https://github.com/Rowena-h/GaeumannomycesGenomics. Boxes outlined in black indicate custom scripts written for this study. **b** Summary of the steps involved in the candidate secreted effector protein (CSEP) prediction.

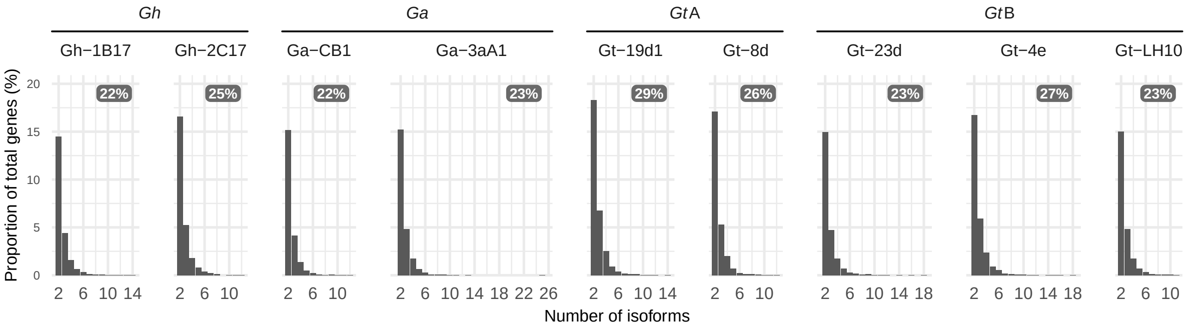

Fig. S2 Histograms showing the proportion of genes with multiple isoforms annotated by REAT. Overall percentage of genes with two or more isoforms are shown in boxes to the top right of each plot.

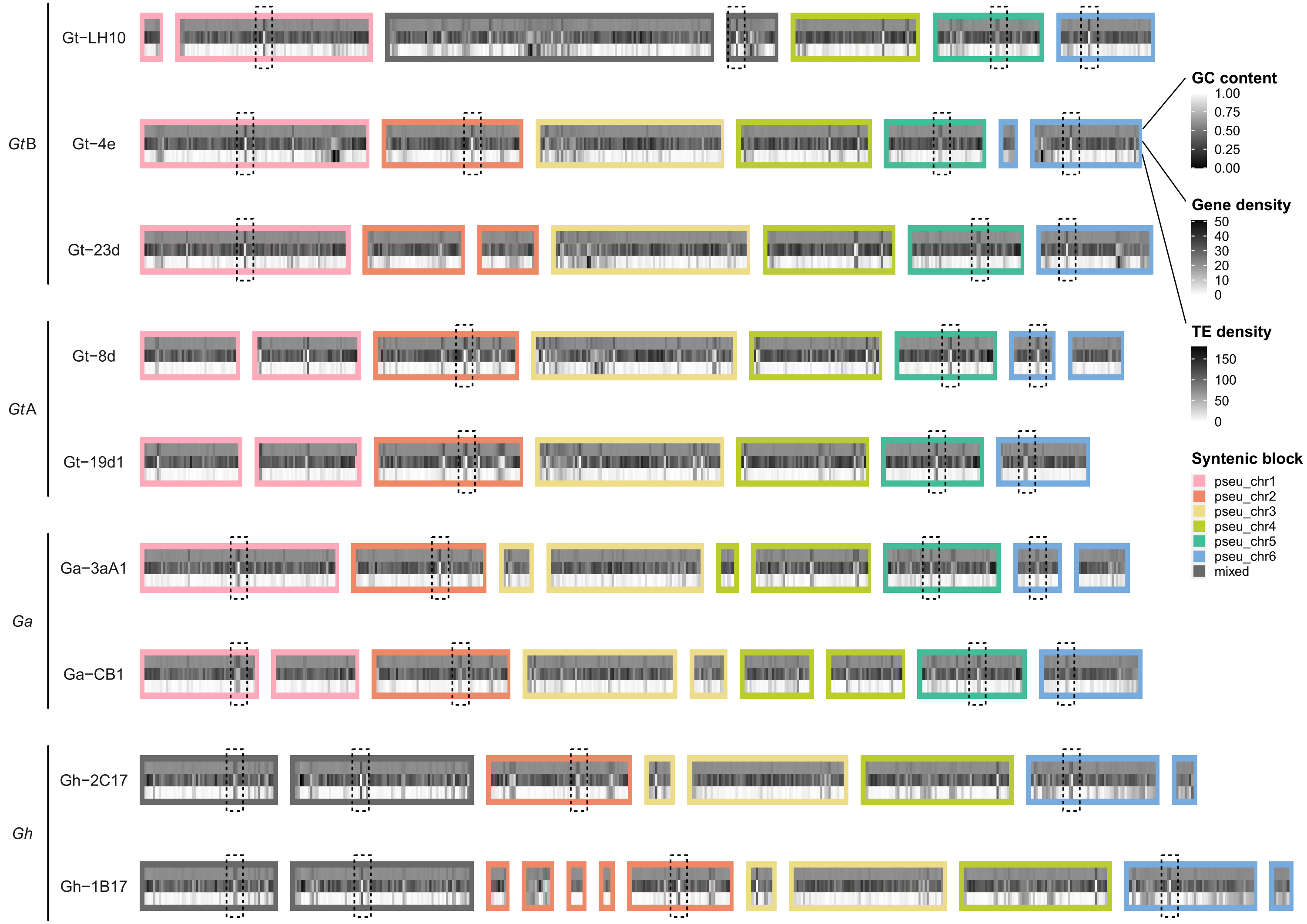

Fig. S3 Putative centromeres identified from GC content, transposable element (TE) density and gene density. All calculations were done in 100,000 bp windows.

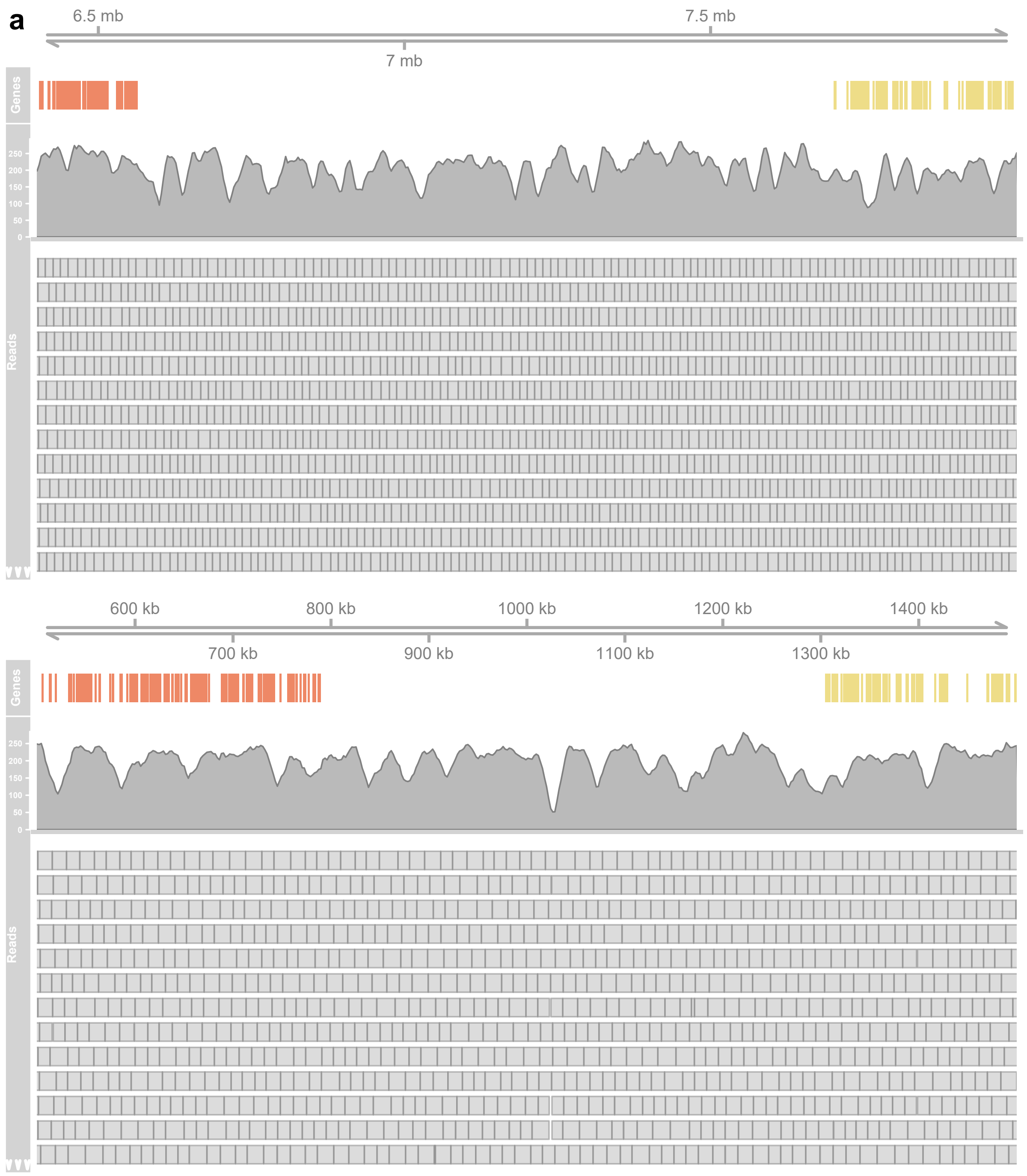

Fig. S4 a Read coverage across the putative translocation in pseudochromosome 2B (top) and pseudochromosome 3B (bottom) in strain Gt-LH10. Grey boxes indicate the location of reads mapped to the assembly. Gene colours match the GENESPACE syntenic blocks in Fig. 2. Note that pseudochromosome 3B has not been inverted as in the GENESPACE plot. ▼

b

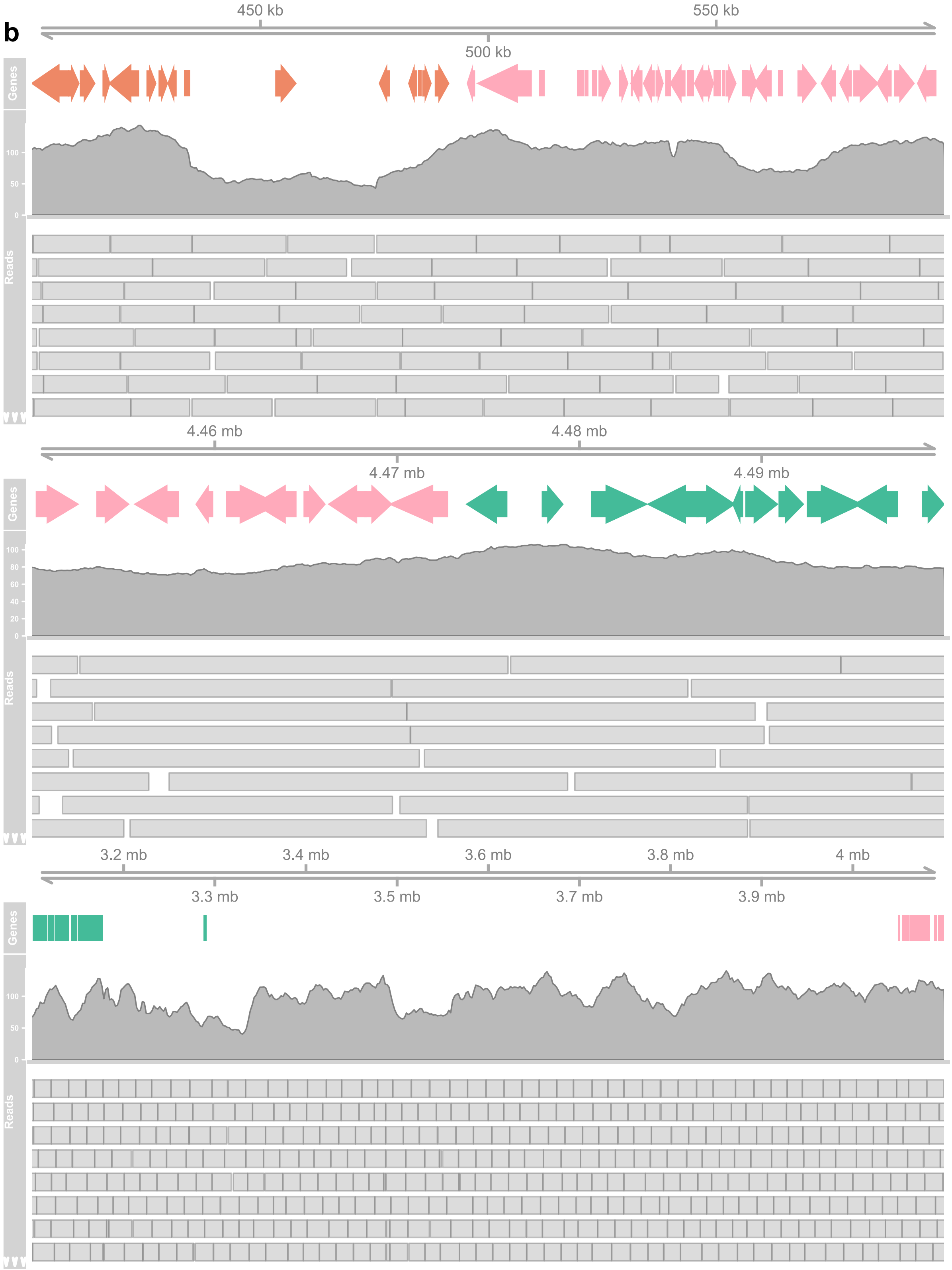

Fig. S4 continued b Read coverage across the putative translocations in pseudochromosome 1B (top and middle) and pseudochromosome 5B (bottom) in strain Gh-1B17. Grey boxes indicate the location of reads mapped to the assembly. Gene colours match the GENESPACE syntenic blocks in Fig. 2. ▼

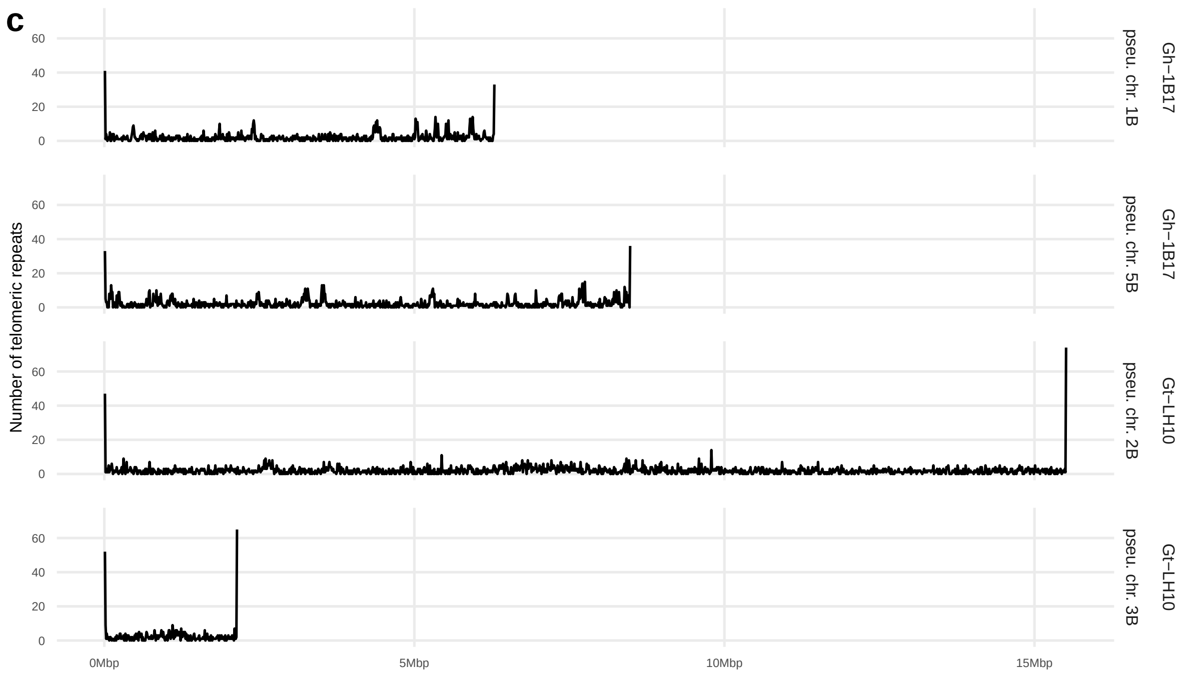

Fig. S4 continued c Telomeric repeats as estimated by tidk across Gh-1B17 pseudochromosomes 1B and 5B and Gt-LH10 pseudochromosomes 2B and 3B.

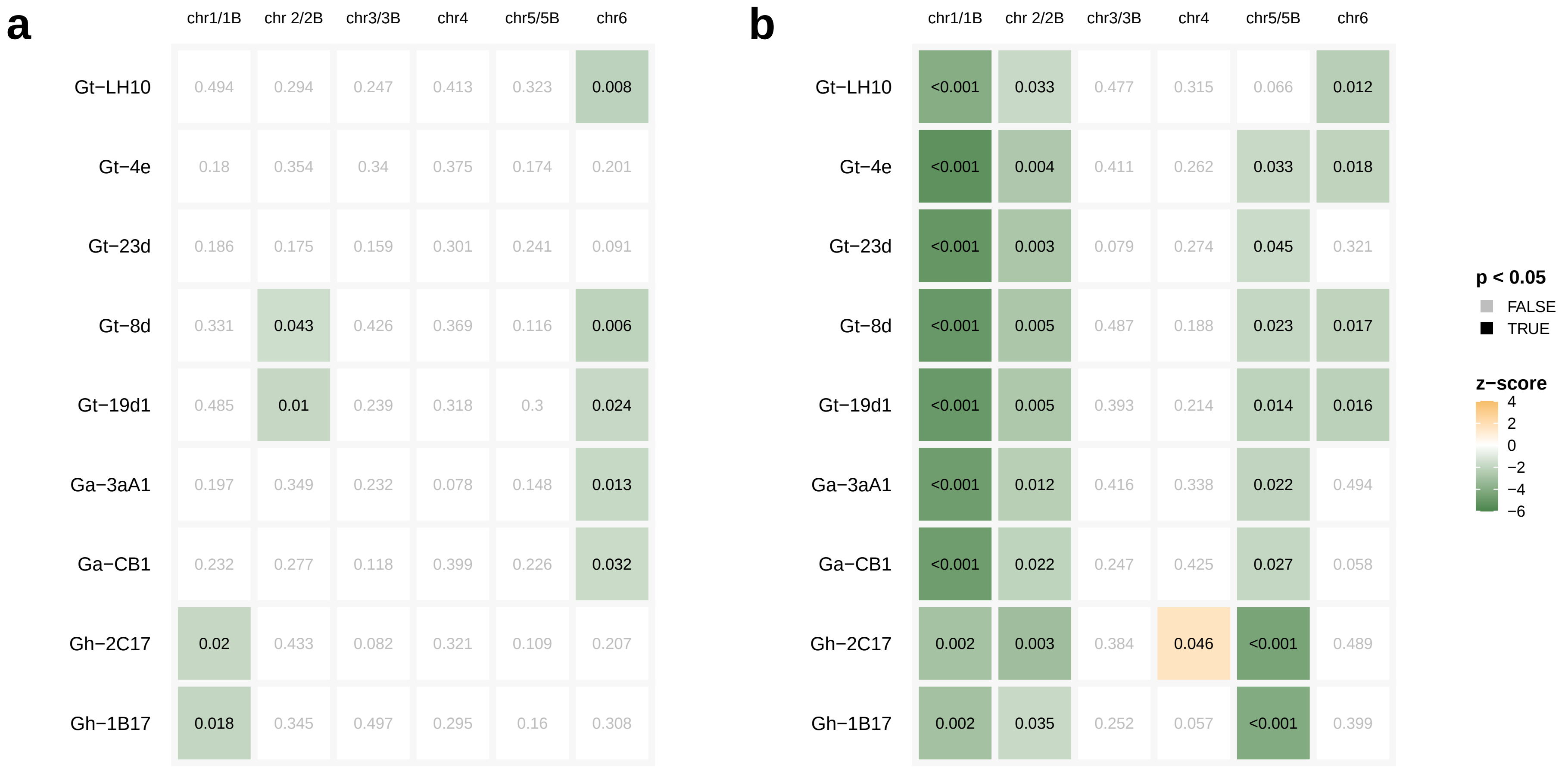

Fig. S5 Matrices of p values from permutation analyses showing whether candidate secreted effector proteins (CSEPs) are significantly closer than expected to **a** transposable elements (TEs) and **b** telomeres. Each test used 1,000 permutations with random resampling of the gene universe for the pseudochromosome. Statistically significant test results ($p < 0.05$) are coloured by z-score, a proxy for 'strength' of the test result, where negative z-score means smaller than expected distance and positive means greater than expected.

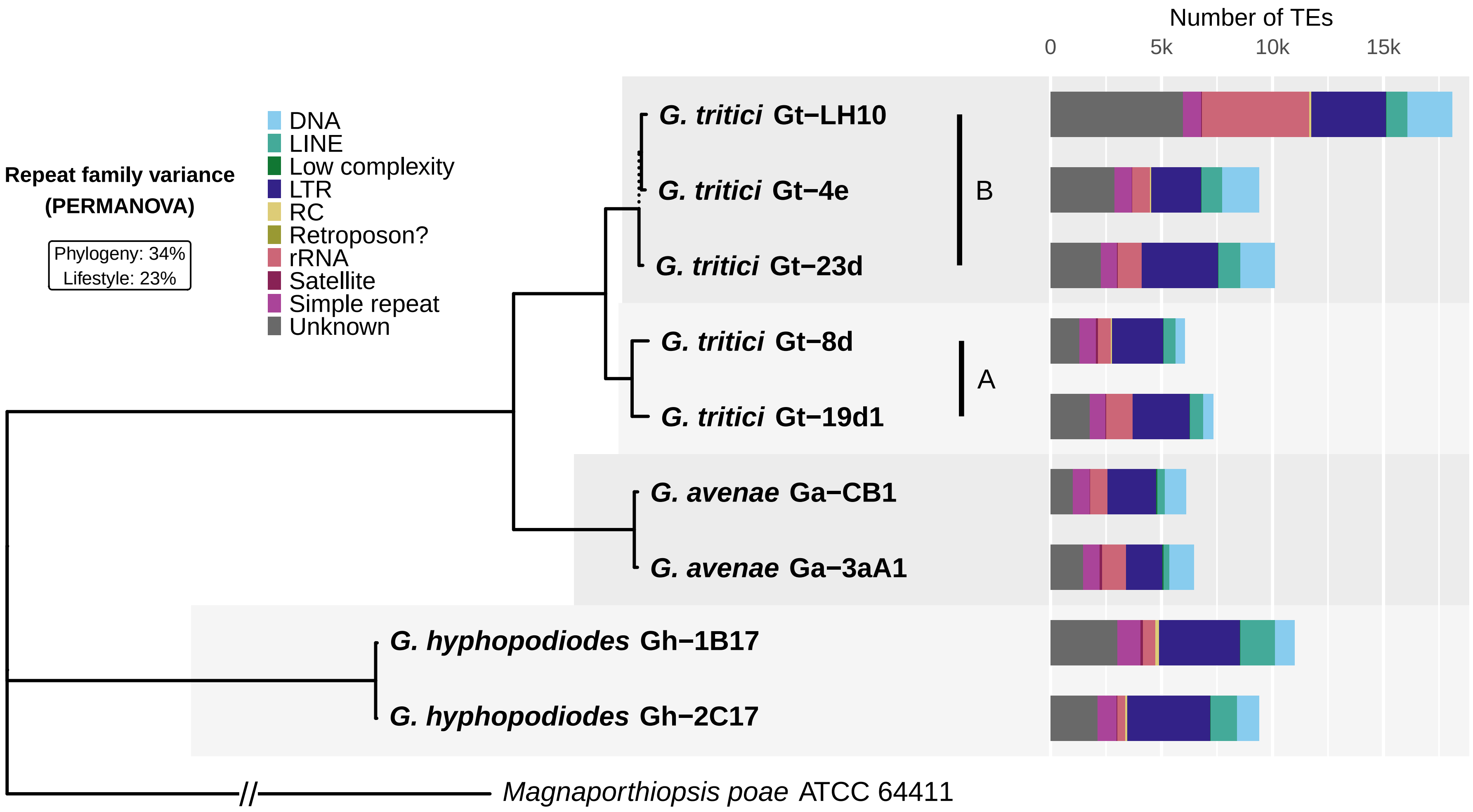

Fig. S6 Total number of transposable elements (TEs) for each strain and their classification as assigned by RepeatMasker.

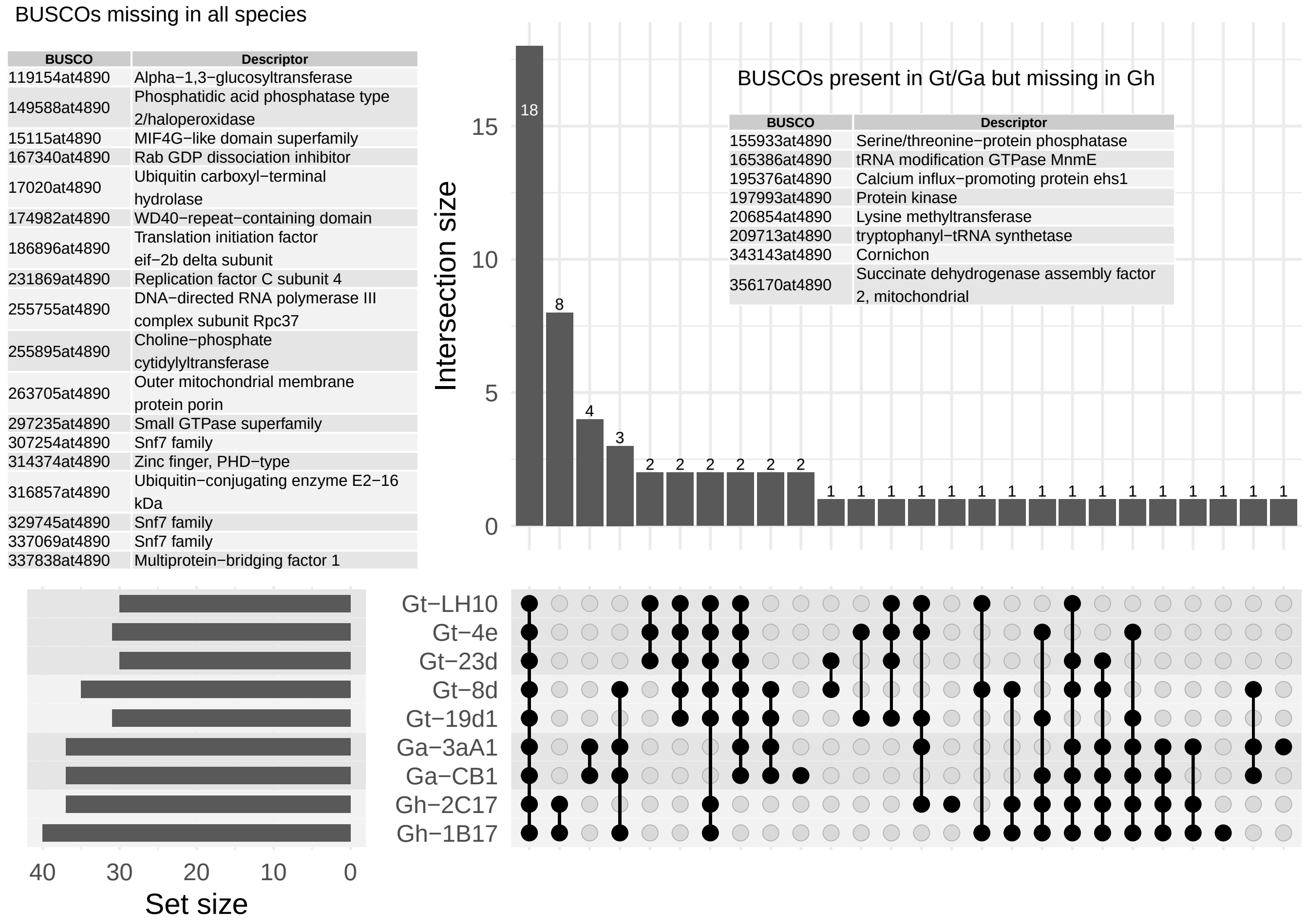

BUSCOs missing in all species

BUSCO	Descriptor
119154at4890	Alpha−1,3−glucosyltransferase
149588at4890	Phosphatidic acid phosphatase type 2/haloperoxidase
15115at4890	MIF4G−like domain superfamily
167340at4890	Rab GDP dissociation inhibitor
17020at4890	Ubiquitin carboxyl−terminal hydrolase
174982at4890	WD40−repeat−containing domain
186896at4890	Translation initiation factor eif−2b delta subunit
231869at4890	Replication factor C subunit 4
255755at4890	DNA−directed RNA polymerase III complex subunit Rpc37
255895at4890	Choline−phosphate cytidylyltransferase
263705at4890	Outer mitochondrial membrane protein porin
297235at4890	Small GTPase superfamily
307254at4890	Snf7 family
314374at4890	Zinc finger, PHD−type
316857at4890	Ubiquitin−conjugating enzyme E2−16 kDa
329745at4890	Snf7 family
337069at4890	Snf7 family
337838at4890	Multiprotein−bridging factor 1

BUSCOs present in Gt/Ga but missing in Gh

BUSCO	Descriptor
155933at4890	Serine/threonine−protein phosphatase
165386at4890	tRNA modification GTPase MnmE
195376at4890	Calcium influx−promoting protein ehs1
197993at4890	Protein kinase
206854at4890	Lysine methyltransferase
209713at4890	tryptophanyl−tRNA synthetase
343143at4890	Cornichon
356170at4890	Succinate dehydrogenase assembly factor 2, mitochondrial

Fig. S7 Upset plot showing the intersections of missing BUSCOs across the *Gaeumannomyces* assemblies. Inset tables list specific BUSCOs missing in all species and missing in just *G. hyphopodioides*.

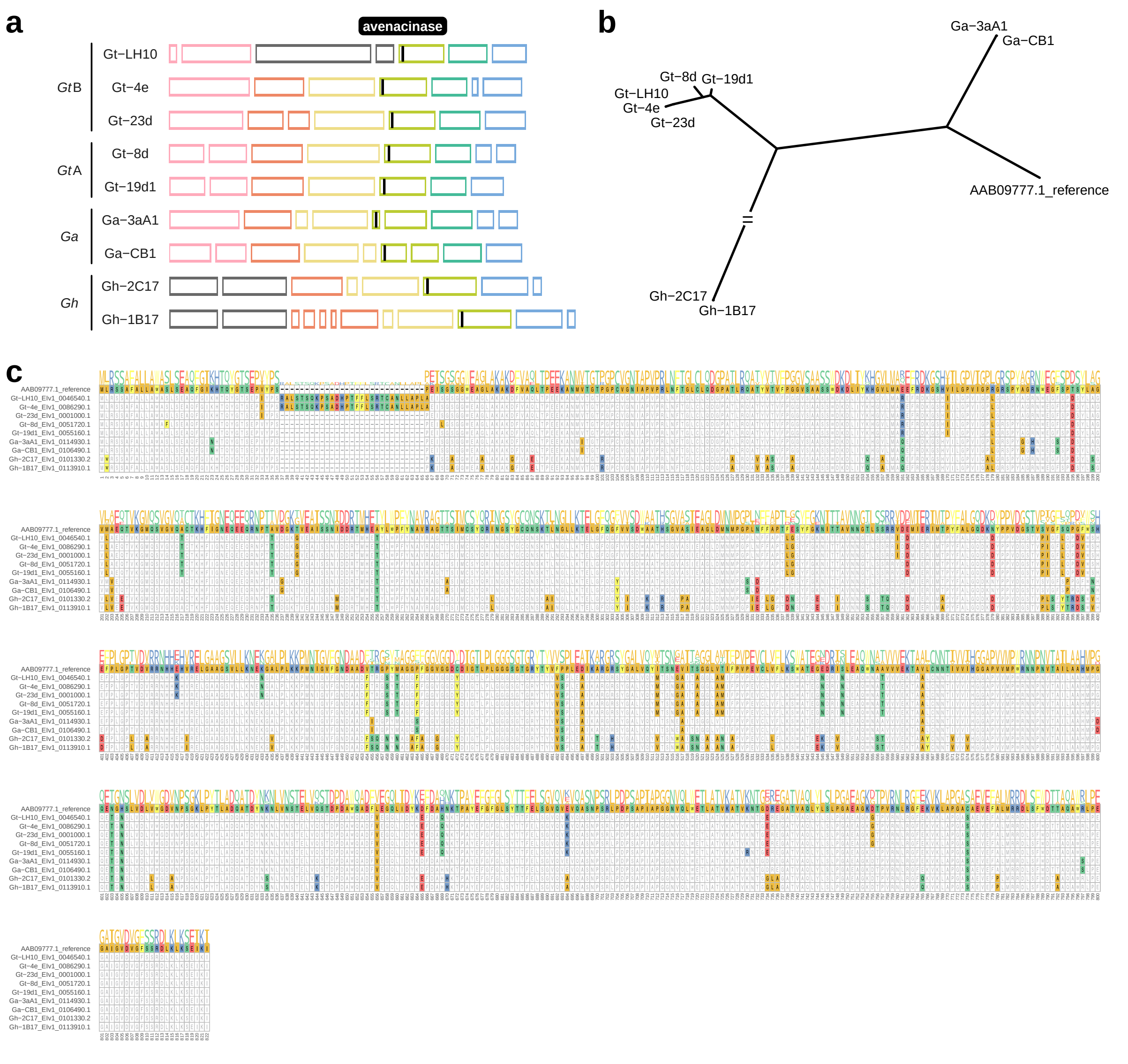

Fig. S8 a Conserved location of the avenacinase gene on pseudochromosome 4. **b** An unrooted RAxML-NG gene tree of the avenacinase gene. **c** Protein sequence alignment of the avenacinase gene, with amino acids coloured if they disagree with the reference sequence in the top row.

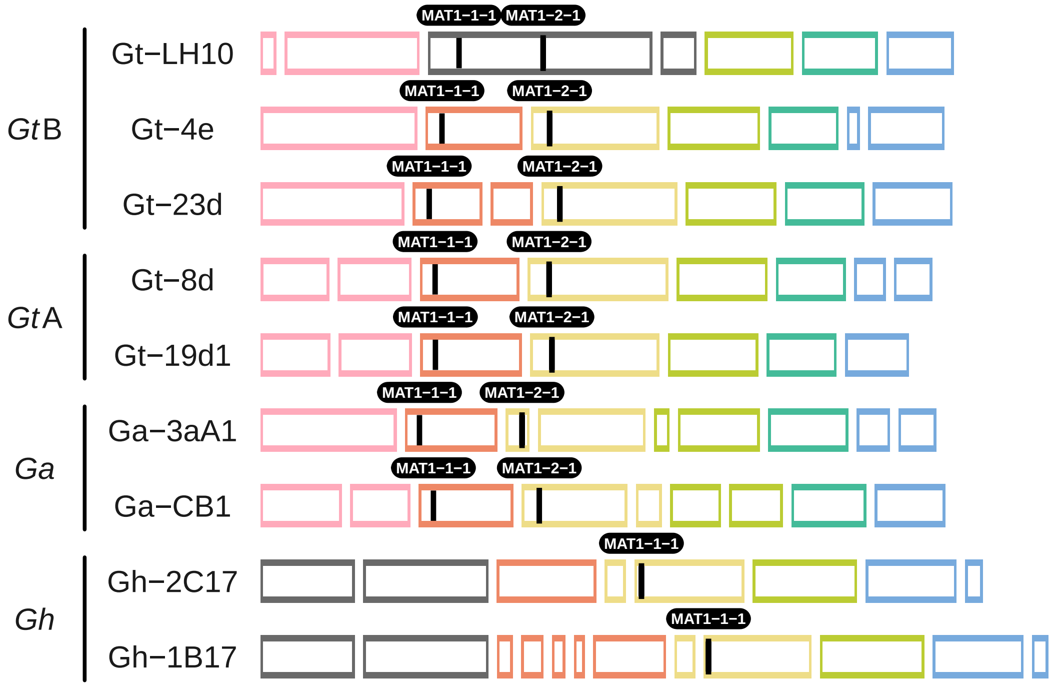

Fig. S9 Location of MAT loci.

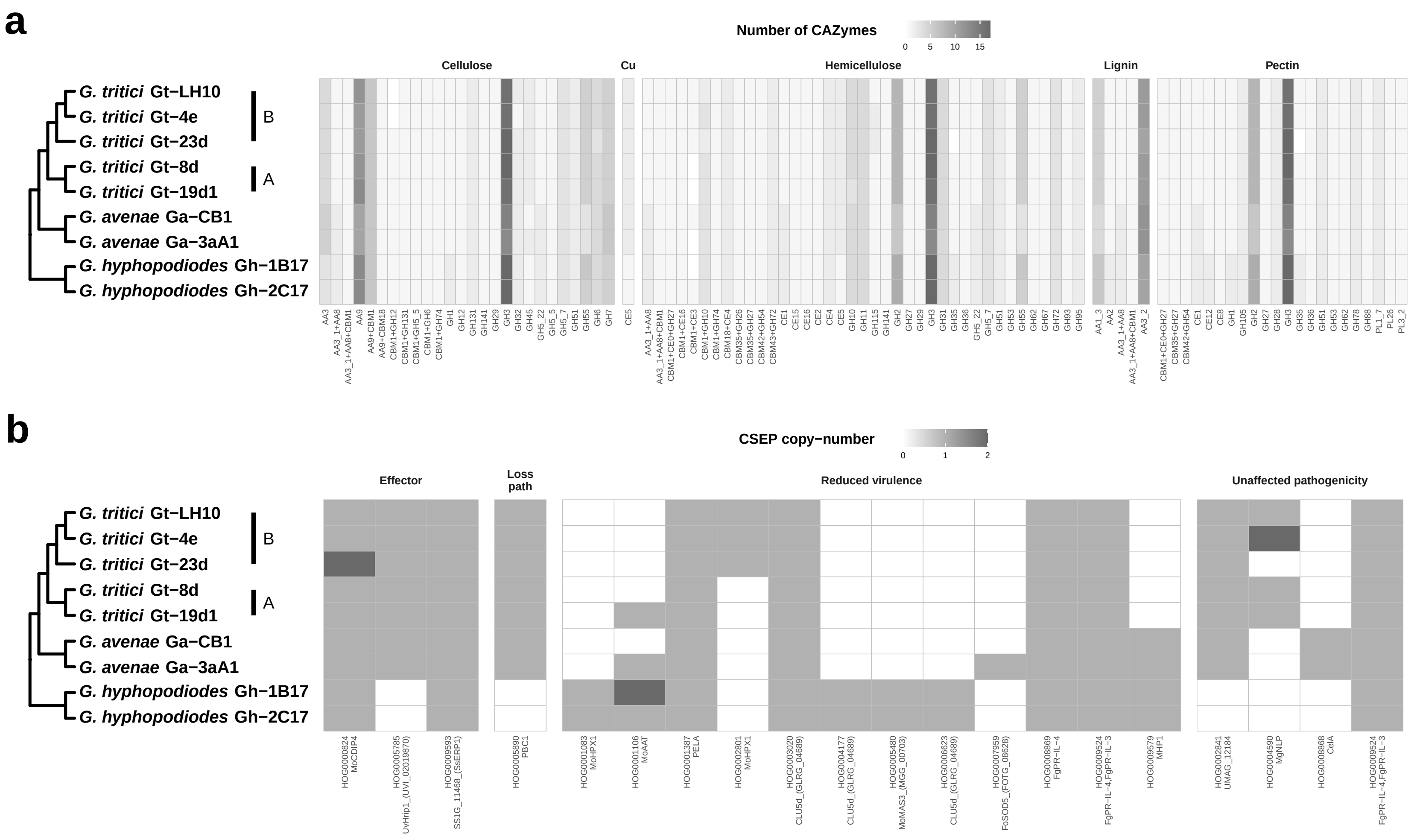

Fig. S10 a Abundance matrix showing number of genes belonging to carbohydrate-active enzyme (CAZyme) families with known plant cell wall substrates (Cu=cutin). **b** Abundance matrix of candidate secreted effector proteins (CSEPs) that could be matched to experimentally verified genes in PHI-base. Genes are grouped based on knockout mutant phenotypes curated in PHI-base (Loss path=loss of pathogenicity). Both the CSEP orthogroup and name of the matched PHI-base gene are show on the x axis. ▼

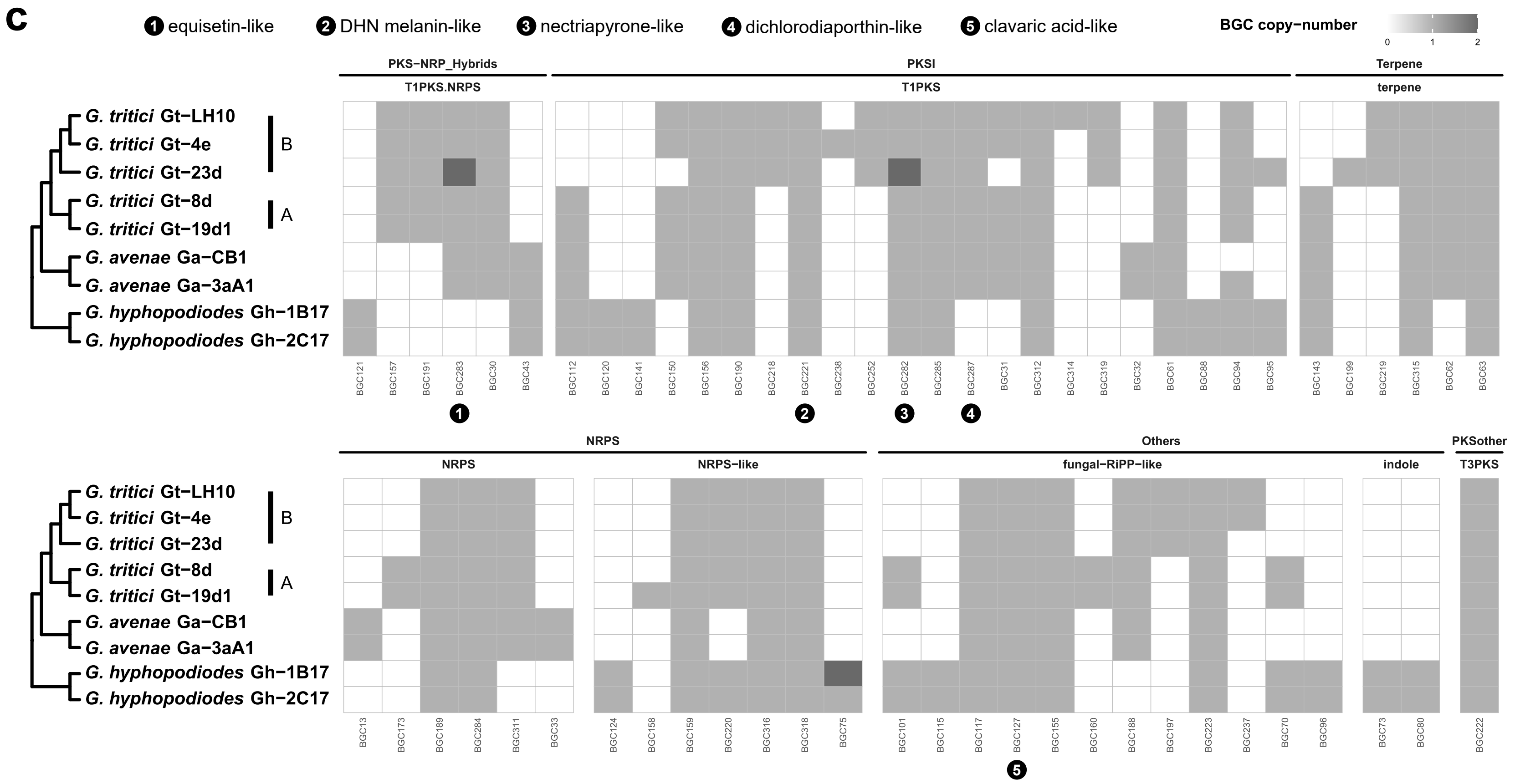

Fig. S10 continued c Abundance matrix of biosynthetic gene clusters (BGCs) belonging to classes assigned by BiG-SCAPE (NRPS=nonribosomal peptide synthetase, PKS=polyketide synthase, PKSI=type 1 polyketide synthase, RiPP=ribosomally synthesised and post-translationally modified peptide, T3PKS=type 3 polyketide synthase). Numbered BGCs were found to have similarity to known clusters in the MIBiG repository.

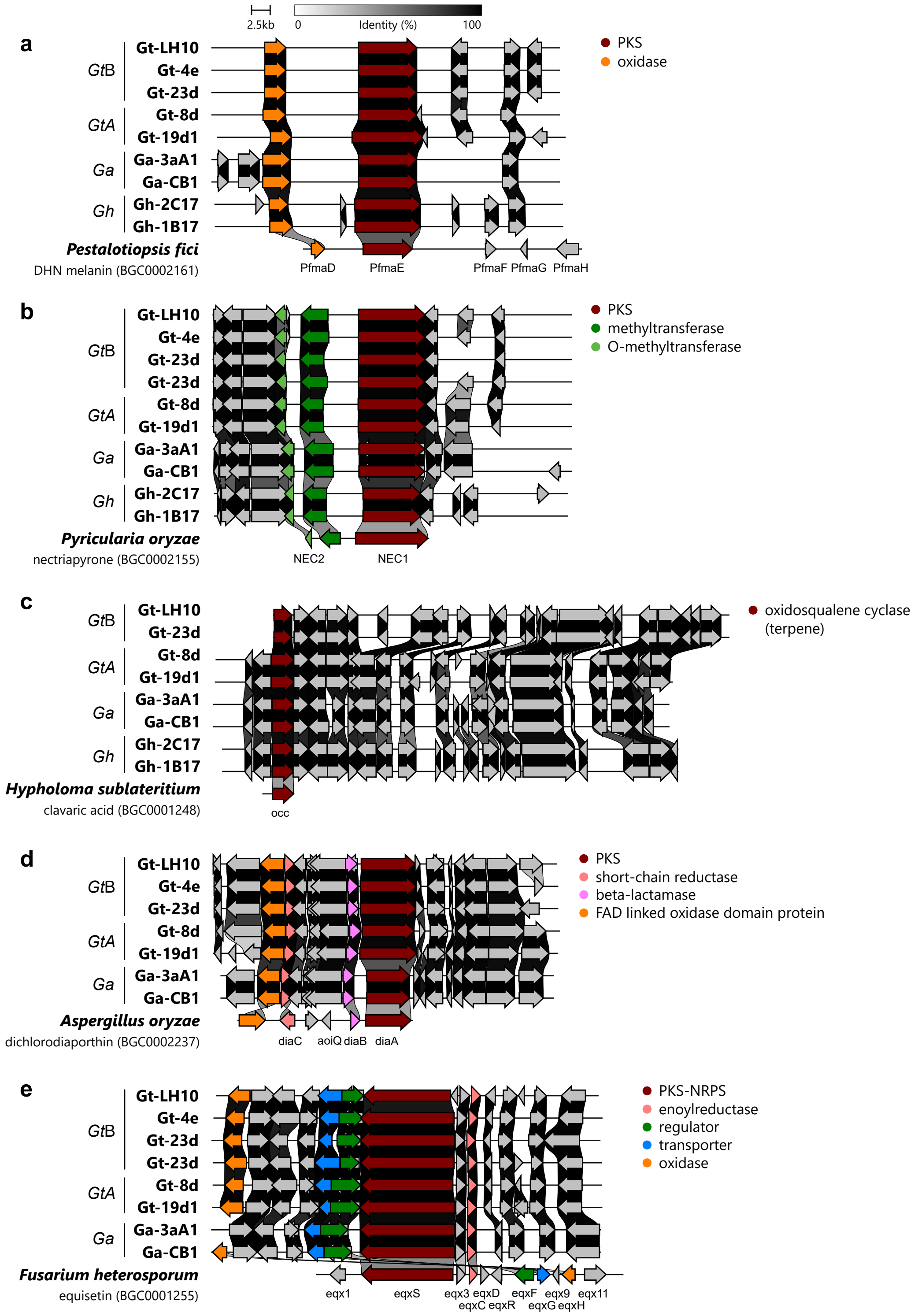

Fig. S11 Schematics of biosynthetic gene clusters (BGCs) that were found to have similarity to known clusters in the MIBiG repository.

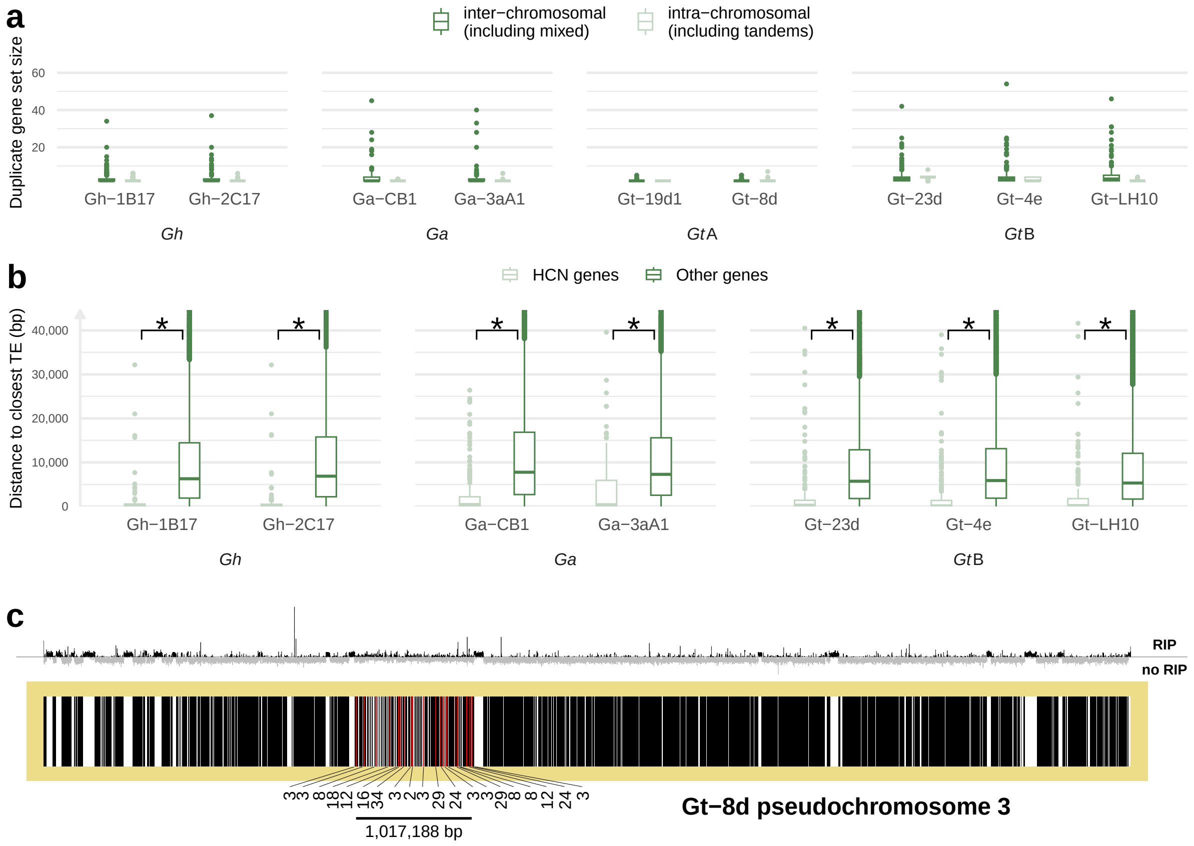

Fig. S12 a Duplicate gene set size for each strain categorised by whether the duplicates only occur on the same pseudochromosome (intra-chromosomal) or at least one duplicate is on a different pseudochromosome (inter-chromosomal). **b** Box plots showing the distance of high copy-number (HCN) genes to the closest transposable element (TE) versus other genes, with outliers > 40,000 bp distant not shown to improve visibility. An asterisk indicates where a Wilcoxon rank sum test found the mean TE distance to be significantly different for HCN genes versus other genes. **c** Region of *Gt* type A strain Gt-8d where low-copy orthologues of genes undergoing copy-number expansions in other lineages cluster on pseudochromosome 3. Red bars indicate the low-copy orthologues, with orthologue ID labelled below, and black bars indicate other genes. The composite RIP index (CRI) calculated in 500 bp windows is indicated above, coloured black when positive and thus indicative of RIP.

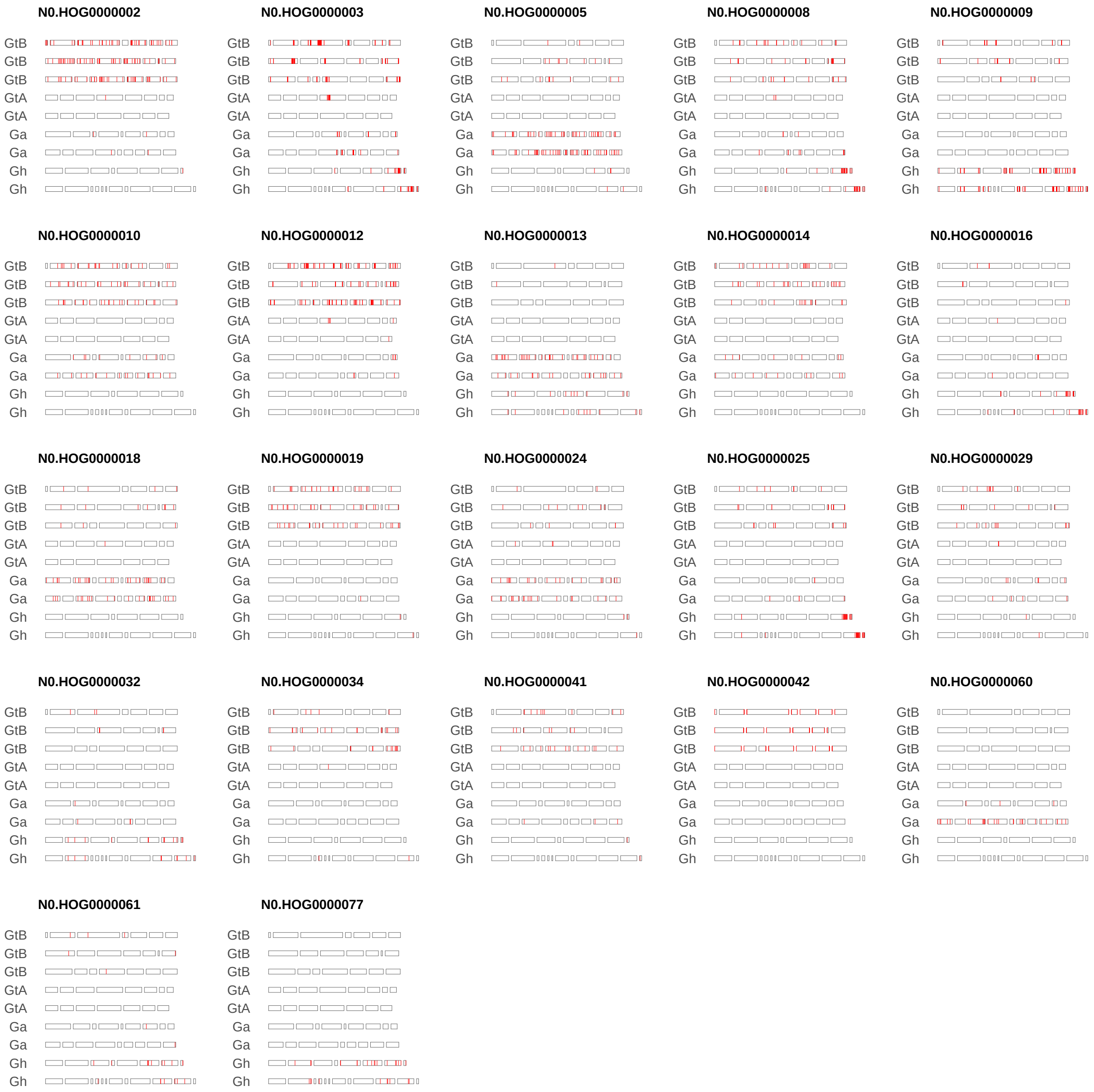

Fig. S13 The location of copy-number expansions for 22 high-copy number genes. Red lines indicate the location of duplicates for the gene, and fragments are ordered syntenically according to GENESPACE (Fig. 2).

a

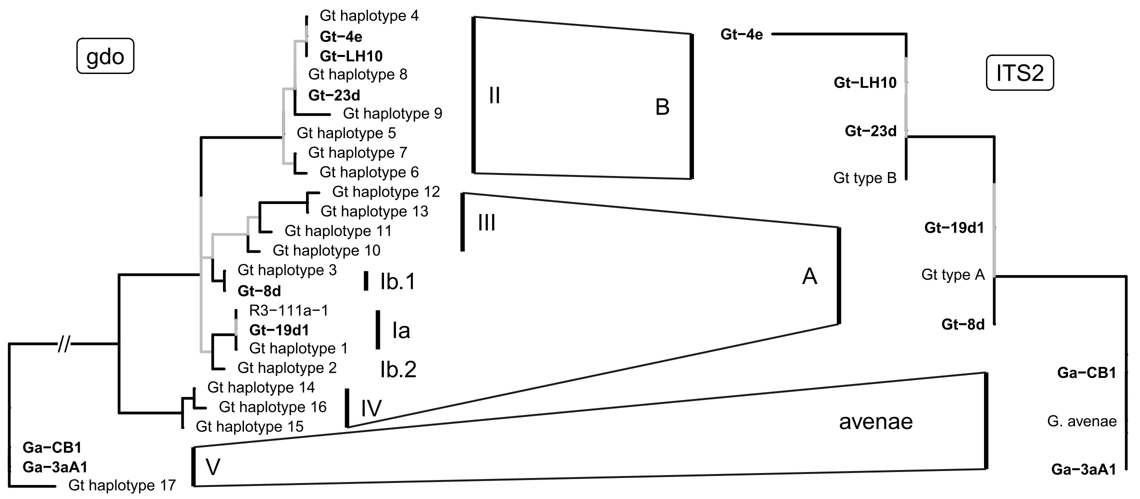

b

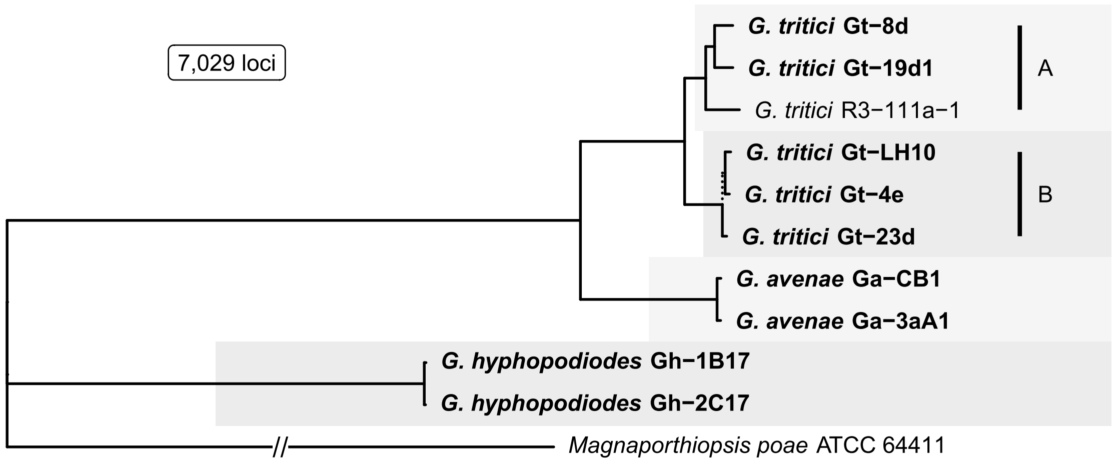

Fig. S14 a Gene trees for the gdo and ITS2 markers and their corresponding genetic groupings of *Gaeumannomyces tritici* (*Gt*) and *G. avenae*. Strains sequenced in this study are in bold and grey branches indicate <70 bootstrap support values. **b** *Gaeumannomyces* species tree with bars indicating type A and type B genetic groups for *Gt*. Strains sequenced in this study are in bold and dashed branches indicate <70 bootstrap support values.

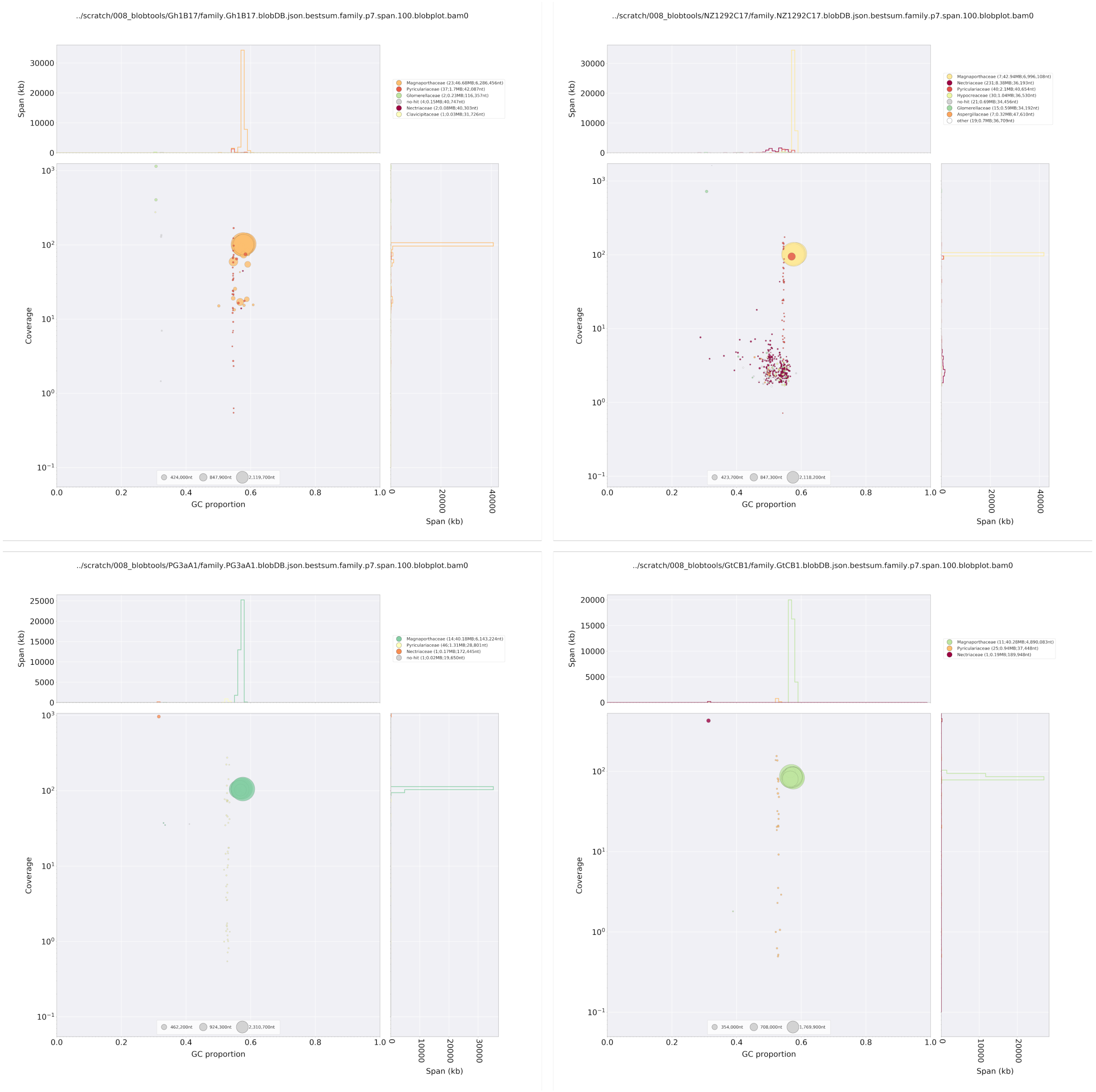

Fig. S15 BlobPlots showing the taxonomic classification of reads based on coverage and GC content for each strain. From top left to bottom right: Gh-1B17, Gh-2C17, Ga-3aA1, Ga-CB1. ▼

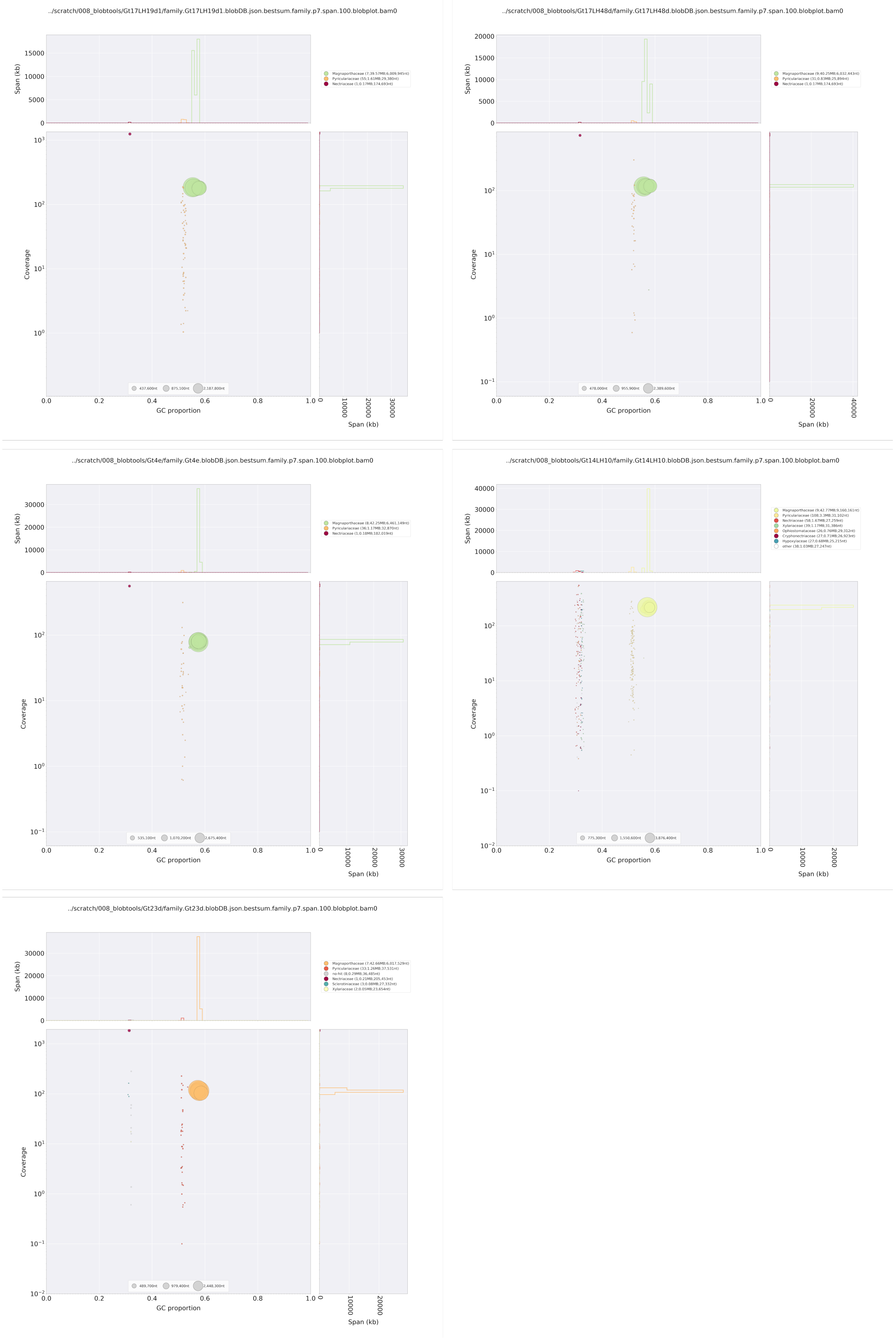

Fig. S15 continued From top left to bottom right: Gt-19d1, Gt-8d, Gt-4e, Gt-LH10, Gt-23d.

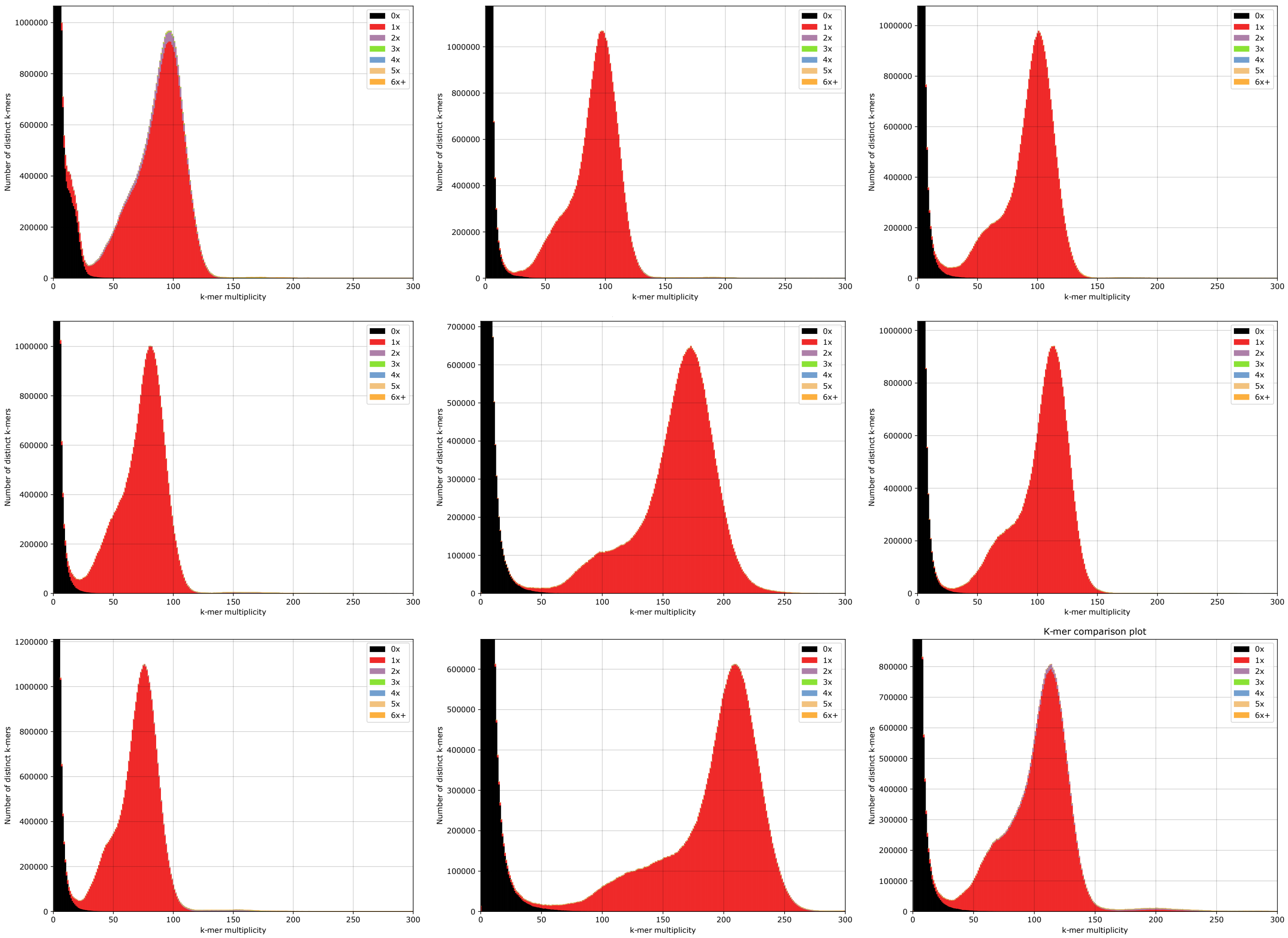

Fig. S16 KAT COMP plots showing content correctness with respect to the input HiFi reads for each strain. From top left to bottom right: Gh-1B17, Gh-2C17, Ga-3aA1, Ga-CB1, Gt-19d1, Gt-8d, Gt-4e, Gt-LH10, Gt-23d.

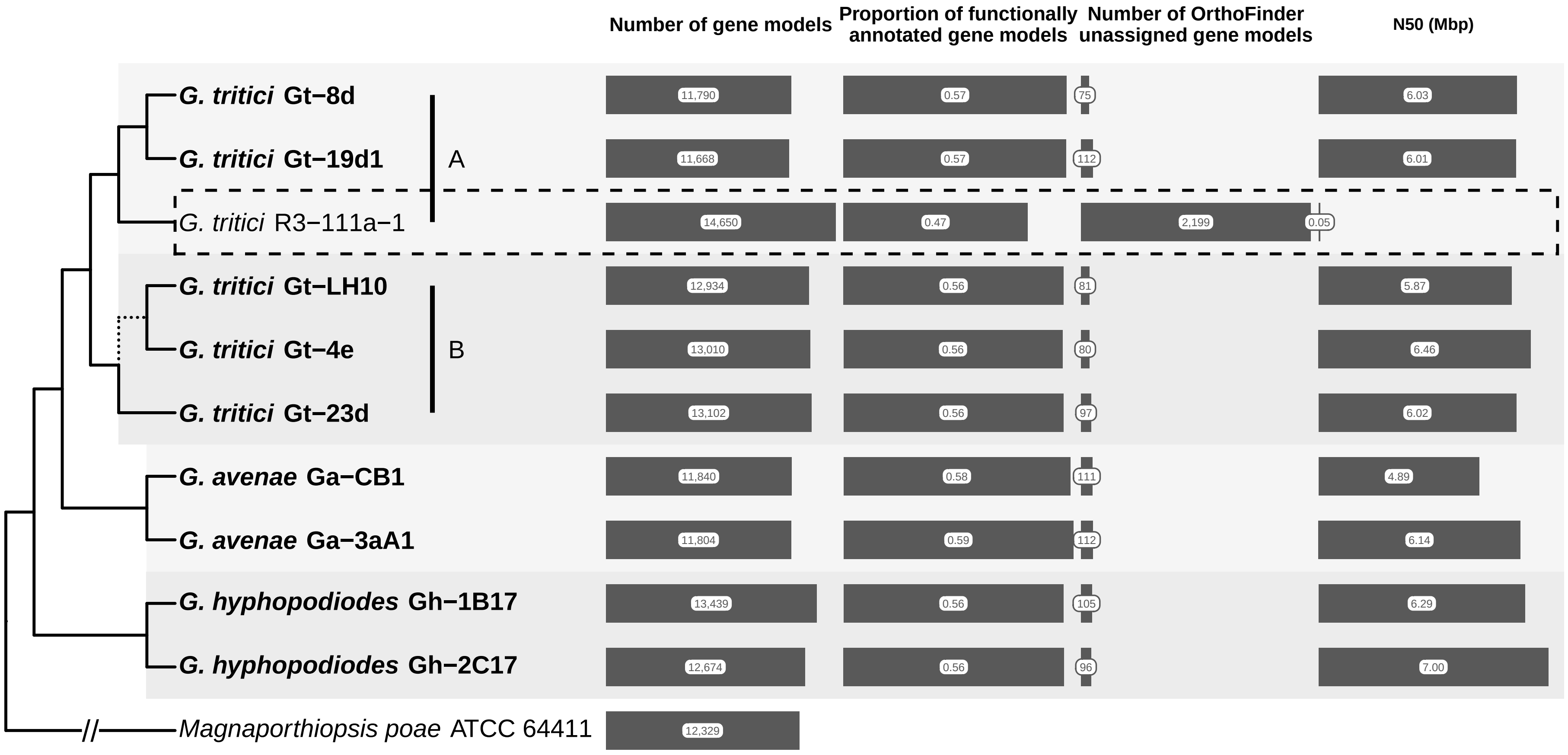

Fig. S17 Comparison of the existing *Gaeumannomyces tritici* R3-111a-1 assembly/annotation (GCF_000145635.1) and the *Gaeumannomyces* assemblies/annotations generated in this study (bold). The proportion of functionally annotated genes refers to results from AHRD.
